# *In Vitro* Characterization of ^177^Lu-DOTA-M5A Anti-Carcinoembryonic Antigen Humanized Antibody and HSP90 Inhibition for Potentiated Radioimmunotherapy of Colorectal Cancer

**DOI:** 10.3389/fonc.2022.849338

**Published:** 2022-03-31

**Authors:** Tabassom Mohajershojai, Preeti Jha, Anna Boström, Fredrik Y. Frejd, Paul J. Yazaki, Marika Nestor

**Affiliations:** ^1^ Department of Immunology, Genetics and Pathology, Uppsala University, Uppsala, Sweden; ^2^ Department of Medicinal Chemistry, Uppsala University, Uppsala, Sweden; ^3^ Ridgeview Instruments AB, Uppsala, Sweden; ^4^ Department of Immunology and Theranostics, Beckman Research Institute, City of Hope, Duarte, CA, United States

**Keywords:** carcinoembryonic antigen (CEA), 177Lu-DOTA-M5A mAb, combination (combined) therapy, multicellular 3D spheroids, colorectal cancer, molecular radiotherapy, HSP90 (heat shock protein 90), onalespib (AT13387)

## Abstract

Carcinoembryonic antigen (CEA) is an antigen that is highly expressed in colorectal cancers and widely used as a tumor marker. ^131^I and ^90^Y-radiolabeled anti-CEA monoclonal antibodies (mAbs) have previously been assessed for radioimmunotherapy in early clinical trials with promising results. Moreover, the heat shock protein 90 inhibitor onalespib has previously demonstrated radiotherapy potentiation effects *in vivo*. In the present study, a ^177^Lu-radiolabeled anti-CEA hT84.66-M5A mAb (M5A) conjugate was developed and the potential therapeutic effects of ^177^Lu-DOTA-M5A and/or onalespib were investigated. The ^177^Lu radiolabeling of M5A was first optimized and characterized. Binding specificity and affinity of the conjugate were then evaluated in a panel of gastrointestinal cancer cell lines. The effects on spheroid growth and cell viability, as well as molecular effects from treatments, were then assessed in several three-dimensional (3D) multicellular colorectal cancer spheroid models. Stable and reproducible radiolabeling was obtained, with labeling yields above 92%, and stability was retained at least 48 h post-radiolabeling. Antigen-specific binding of the radiolabeled conjugate was demonstrated on all CEA-positive cell lines. Dose-dependent therapeutic effects of both ^177^Lu-DOTA-M5A and onalespib were demonstrated in the spheroid models. Moreover, effects were potentiated in several dose combinations, where spheroid sizes and viabilities were significantly decreased compared to the corresponding monotherapies. For example, the combination treatment with 350 nM onalespib and 20 kBq ^177^Lu-DOTA-M5A resulted in 2.5 and 2.3 times smaller spheroids at the experimental endpoint than the corresponding monotreatments in the SNU1544 spheroid model. Synergistic effects were demonstrated in several of the more effective combinations. Molecular assessments validated the therapy results and displayed increased apoptosis in several combination treatments. In conclusion, the combination therapy of anti-CEA ^177^Lu-DOTA-M5A and onalespib showed enhanced therapeutic effects over the individual monotherapies for the potential treatment of colorectal cancer. Further *in vitro* and *in vivo* studies are warranted to confirm the current study findings.

## Introduction

Gastrointestinal (GI) cancers are a collective term describing cancers that affect the digestive system. The most common diagnosed GI cancers are colorectal cancer, gastric cancer, and liver cancer (1). Colorectal cancer is the third most commonly diagnosed cancer and the second cause of cancer death worldwide (2). Carcinoembryonic antigen (CEA) is a well-known cancer-associated biomarker, which has been found in several solid tumors including GI and breast cancers (3–5). Consequently, CEA has been used as a target for cancer diagnosis, research, and targeted therapy (6–8).

High expression of CEA on the basolateral surface of colorectal cancer cells has made it a suitable target for colorectal cancer-targeted therapies, including antibody–drug conjugates and radionuclide therapies (7, 9–14). Despite a large number of *in vivo*, preclinical, and clinical studies on targeting CEA, the underlying cellular and molecular mechanisms involved in tumor suppression and metastasis inhibition by targeting CEA are not fully clear. CEA is a glycosylphosphatidylinositol (GPI) membrane-bound protein with a very slow rate of internalization (15) with no direct downstream signaling pathway but can influence other pathways indirectly such as the transforming growth factor (TGF)-β pathway (16).

Radioimmunotherapy (RIT) is a promising approach for the treatment of many cancers. Targeting an antigen that is specifically and highly expressed on cancer cells can be a suitable way to preferentially deliver therapeutic radionuclides to tumors. The efficiency of RIT requires a specific and long enough interaction between antigen and radiolabeled conjugate (antibody) in order to deliver an optimal tumoricidal radiation dose. RIT has been demonstrated as an effective therapy for hematological malignancies, whereas the effects on solid tumors have been more limited due to characteristics such as antigen heterogenicity, drug penetration limitations, tumor location, limited vascularization of the tumor, and rapid cell proliferation. Despite these factors, RIT has still shown promise for some solid tumors such as colorectal cancers (17–19).

Several CEA-targeting antibodies have previously been labeled with various radionuclides and explored *in vitro*, *in vivo*, and in clinical trials, demonstrating promising results (10–12, 20, 21). T84.66 is a well-characterized anti-CEA murine IgG_1_ monoclonal antibody (mAb) that does not cross-react with other members of the *CEA* gene family and has successfully been used for ^111^In imaging of patients with primary colorectal adenocarcinomas (22, 23). To avoid human immune responses, the T84.66 mAb was reconstituted to a chimeric version (cT84.66), where it was conjugated with the bifunctional chelates Diethylenetriamine Pentaacetate (DTPA) and Dodecane Tetraacetic Acid (DOTA) and radiolabeled with ^111^In and ^90^Y. The cT84.66 hT84.66, M5A was evaluated in extensive RIT clinical studies (21 trials with over 250 patients) as a single agent or in combination with standard of care chemotherapy agents (24). The clinical studies demonstrated that the antibody was well tolerated and feasible to use clinically in combination with chemotherapy agents (7, 24–29). Recently, a humanized version of T84.66 (hT84.66, M5A) was developed to lower potential immunogenicity (30). A Phase I ^90^Y-DOTA-M5A RIT study was conducted in patients with advanced CEA-expressing tumors, and some therapeutic effects were observed, suggesting a therapeutic potential of this agent (11).

In recent years, ^177^Lu has been established as a promising radionuclide for targeted radionuclide therapies, of which cost-effectiveness, availability, well-suited physical and biological half-life, and desired energy in order to minimize off-target effects are some of the contributing factors (31). However, the anti-CEA humanized mAb M5A has not previously been assessed with ^177^Lu and subsequent use for RIT. Therefore, the first aim of this study was to optimize ^177^Lu labeling of M5A and characterize the conjugate *in vitro*.

Other interesting targets for cancer therapy are the heat shock proteins (HSPs), which are involved in many oncogenic pathways. HSPs are a large family of molecular chaperones involved in protein folding, maturation, and degradation. Under stress conditions such as heat shock, oxidative stress, and any other stress events leading to protein damages, HSP responses are critical (32). HSP90 is a known cellular protein acting on numerous hallmarks of cancer, such as cell proliferation, apoptosis, and invasiveness. Particularly, HSP90 plays a key role in regulatory protein folding pathways, transcription factors, and cellular kinases (33). On a molecular level, HSP90 influences, e.g., Receptor Tyrosine Kinases (RTKs), Phosphoinositide 3-Kinase (PI3K)/AKT, and Mitogen-Activated Protein Kinase (MAPK)/Extracellular-Signal-Regulated Kinase (ERK) pathways. It can partially regulate Wnt and TGF-β signaling pathways as well. RTK blockade, e.g., toward epidermal growth factor receptor (EGFR) or vascular endothelial growth factor receptor (VEGFR), combined with HSP90 inhibitors demonstrated therapeutic potential in preclinical and clinical colorectal studies (34–36). High HSP90 expression is associated with invasion, metastasis, and poor prognosis of colorectal cancers and gastric cancers (37, 38). Therefore, inhibition of HSP90 may be a feasible strategy to increase cytotoxicity and suppress protection mechanisms of cancer cells influencing different pathways simultaneously (39). Though there are plenty of HSP90 inhibitors in clinical trials, none is approved for cancer monotherapy due to toxicity and difficult formulation of the first-generation of inhibitors (40). Onalespib (AT13387) is a second-generation HSP90 inhibitor indicated to have better solubility and minimal hepatotoxicity as compared to the first-generation HSP90 inhibitors (e.g., 17-AAG and 17-DMAG) in both preclinical and clinical trials (41). Onalespib has been used alone and in combination with chemotherapy drugs and radiation for solid tumors in preclinical and clinical studies (42–44). Radiosensitizing features have been demonstrated *in vivo* when onalespib was combined with external beam radiation in e.g., colorectal cancer models (45) and with targeted radionuclide therapy using ^177^Lu-DOTATATE in neuroendocrine cancer models (46). Thus, the radiosensitizing property of onalespib in addition to its aforementioned inhibition on cellular pathways makes it a highly interesting candidate to combine with ^177^Lu-DOTA-M5A in colorectal cancer treatment. Thus, the second aim of this study was to evaluate the effects of HSP90 inhibition in colorectal cancer models *in vitro* and to assess the radiosensitizing potential in combination with ^177^Lu-DOTA-M5A.

To conclude, the aim of the present study was to develop and characterize a ^177^Lu-DOTA-M5A conjugate and assess if therapeutic effects could be further potentiated through HSP90 inhibition. Therapeutic and molecular effects of both monotreatments and the combination treatments were assessed in a panel of colorectal cancer cell lines using two-dimensional (2D) models, and three-dimensional (3D) multicellular spheroid models.

## Materials and Methods

### Cell Culture and Maintenance

The human GI CEA-positive cell line SNU1544 [colon adenocarcinoma, doubling time 42 h (47)] obtained from Korean Cell Line Bank (KCLB) was cultured in RPMI [Biowest, MO, USA, containing 25 nM N-2-Hydroxyethylpiperazine-N'-2-Ethanesulfonic Acid (HEPES)] supplemented with 10% (v/v) fetal bovine serum (FBS; Sigma Aldrich, MO, USA) that was heat inactivated at 56°C for 30 min. MKN45 cell line [gastric adenocarcinoma, doubling time 60 h (48)] obtained from German Collection of Microorganisms and Cell Culture (DSMZ) was cultured in RPMI supplemented with 20% FBS. HT55 cell line [colon carcinoma, doubling time 28 h (49)] obtained from European Collection of Authenticated Cell Culture (ECACC) was cultured in Minimum Essential Medium (MEM) (Biowest, MO, USA) supplemented with 20% FBS. LS174T cell line [colon adenocarcinoma, doubling time 26 h (50)] obtained from ECACC was cultured in the same media as HT55 cell line except supplemented with 10% FBS. The human colon adenocarcinoma HT29 [doubling time 24 h (51)] obtained from American Type Culture Collection (ATCC) was cultured in McCoy’s (Biowest, MO, USA) supplemented with 10% FBS. All media were supplemented with L-glutamine (Biochrom GmbH, 2 mM) and antibiotics (100 IU penicillin and 100 µg/ml streptomycin, Biochrom GmbH, Germany). Monolayer cultures were grown in tissue culture flasks (VWR, PA, USA) and incubated in an atmosphere containing 5% CO_2_ at 37°C. After reaching 70%–80% confluency, cell passaging was performed using Trypsin-EDTA (Biochrom GmbH, Germany). Differences in cell culture media and supplements may to some extent impact cellular processes. In the present study, all studied cell lines were grown in the culture conditions recommended by the suppliers.

### Anti-Carcinoembryonic Antigen M5A Monoclonal Antibody and Onalespib

The hT84.66-M5A (M5A) mAb is a humanized IgG_1_ mAb derived from the murine T84.66 mAb by Complementary Determining Region (CDR) grafting based on structure design (30). The M5A mAb was conjugated with NHS-DOTA as previously described (52). Briefly, diafiltration was used for buffer exchange, conjugation, and concentration of DOTA-M5A mAb using an Amicon ultrafiltration stirred cell 8300 under vacuum (MilliporeSigma, Burlington, MA, USA). The M5A mAb (5 mg, 5 mg/ml) was equilibrated in 10 diavolumes of conjugation buffer (100 mM sodium bicarbonate buffer, 5 mM DTPA, pH 8.5). A 10-M excess of DOTA-NHS ester dissolved in the conjugation buffer (0.282 mg, 10 mg/ml, Macrocylics, Plano, TX, USA) was added to the M5A mAb in a total volume of 1 ml and allowed to stir for 1 h at room temperature. The reaction was buffer exchanged with 18 diavolumes of post-conjugation buffer (250 mM ammonium acetate, pH 7.0) and filtered under sterile condition (0.2 µm, Thermo Fisher Scientific, Waltham, MA, USA). All buffers were prepared using Ultra Trace water (Thermo Fisher Scientific) and sterile filtered prior to use. The resulting DOTA-M5A conjugate was characterized by measurement of protein concentration (A_280 nm_), Isoelectric Focusing (IEF) analysis (Criterion IEF pre-cast gel, pH 3-10, Bio-Rad Laboratories, Hercules, CA, USA), High Performance Liquid Chromatography (HPLC)-size exclusion chromatography (HPLC-SEC, Superdex-200 10/300 column, Cytiva, Marlborough, MA, USA), and the quadrupole time-of-flight (QTOF) mass spectrometry (model 6520, Agilent Technologies, Santa Clara, CA, USA; **Supplementary Figure S1**).

For immunoreactivity, DOTA-M5A mAb was radiolabeled with ^64^Cu with 97.5% radiolabeling yield as confirmed by instant thin-layer chromatography (ITLC, mAb stays at origin, ^64^Cu-DTPA migrates to front). The purity of ^64^Cu-DOTA-M5A (r_t_ = 40.55 min) was analyzed by HPLC-SEC (Superdex 200 10/300 column, with an in-line OD_280_ UV detector and radiodetector, 0.5 ml/min, PBS-1% HSA, isocratic; **Supplementary Figure S2**). The radioconjugate ^64^Cu-DOTA-M5A (200,000 cpms) was incubated with 20× molar excess of soluble CEA for 30 min at room temperature. The reaction mixture was then analyzed on HPLC-SEC Superdex 200, monitoring for a molecular size shift of the radioactive mAb to an increased molecular size (**Supplementary Figure S2**).

The lyophilized onalespib (AT13387) (Selleckchem, TX, USA) was dissolved in Dimethyl Sulfoxide (DMSO) to a stock concentration of 61.0471 mM and stored at -20°C until required for the experiments. Onalespib was thawed and diluted further in cell media for cellular treatments.

### ^177^Lu Radiolabeling and Purification

Prior to ^177^Lu labeling, DOTA-M5A mAb was buffer exchanged to either sodium acetate (0.2 M, pH 5.5) or ammonium acetate (0.1 M, pH 5.5) using spin column (Amicon Ultra-0.5 ml, Merck, Germany). The nominal molecular weight cutoff (NMWCO) of the spin column was 3 kDa. Spin column was rinsed once with NaOH (0.1 M, 500 µl, 14,000 rpm, 10 min, 22°C) followed by subsequent rinsing with the sodium acetate or ammonium acetate buffer under similar conditions. The desired amount of DOTA-M5A was loaded on a rinsed column, its volume was made up to 500 µl with buffer, and the sample was rinsed as mentioned before. The concentrated antibody was dissolved in the desired buffer volume and collected in a new tube. The concentration of final DOTA-M5A was measured using Nanodrop (Denovix DS-11 Spectrophotometer, DE, USA). Molecular mass of conjugate was approximated to 150 kDa (**Supplementary Figure S1**).

^177^Lu radiolabeling was optimized (**Supplementary Figures S3** **,** **S4**), and the optimized conditions were then applied for ^177^Lu labeling of DOTA-M5A. Briefly, mixture of ^177^Lu (15 MBq, in 0.04 M HCl, ITG GmbH) and DOTA-M5A (50 µg) dissolved in sodium acetate (0.2 M, pH 5.5) or ammonium acetate (0.1 M, pH 5.5) was stirred on the thermoshaker (TS-100C Smart, Biosan, Lativa) at 42°C for 1 h (350 rpm), yielding the final specificity activity of 300 kBq/µg (≥90% radiochemical yield). The extent of radiolabeling was assessed with ITLC (Biodex Medical Systems) using citric acid (0.2 M, pH 5.5) as a mobile phase, and the radiolabeling yield was quantified using phosphoimager (BAS-1800II, Fujifilm). Radiolabeling purity (≥99.9%) of ^177^Lu-DOTA-M5A was assessed using HPLC-SEC (in-line OD_280_ UV-detector and radiodetector: Chromater Alpha; Phenomenex, BioSep-SEC-s3000, LC Column 300 × 7.8 mm, 1.0 ml/min, 0.1 M phosphate buffer, pH 6.8, isocratic; **Supplementary Figure S4**: UV-detection, r_t_ = 8.65 min, radio-detector, r_t_ = 8.75 min). The ^177^Lu-DOTA-M5A was purified as and when required using spin column (Amicon Ultra-0.5 ml, Merck, Germany, 3 kDa NMWCO).

### Stability Assays

The stability of ^177^Lu-DOTA-M5A was assessed in mouse serum and in EDTA condition, respectively. Briefly, for stability in serum, ^177^Lu-DOTA-M5A (10 µg) was added to individual Eppendorf tubes containing either PBS (80 μl) or mixture of mouse serum and PBS (1/1, v/v, total volume of 80 µl). For the EDTA challenge test, 500-M excess of EDTA (dissolved in metal-free water) was added to ^177^Lu-DOTA-M5A. Metal-free water was added to ^177^Lu-DOTA-M5A in a separate tube, as control. Samples were stirred well and placed on thermoblock for 48 h at 37°C. Stability was analyzed for the overall radiolabeling yield using ITLC and quantified as mentioned previously at 1 and 48 h post-radiolabeling.

### Two-Dimensional lysates and Western Blot Analyses

To evaluate potential onalespib effect on the CEA expression on CEA-positive cell lines, a defined number of cells were seeded in T25 flasks (1 × 10^6^ SNU1544 cells/flask, 0.7 × 10^6^ LS174T cells/flask, and HT55 cells/flask), incubated in an atmosphere containing 5% CO_2_ at 37°C and treated at 70% confluency. SNU1544 cells were treated with 50, 150, 350, and 700 nM of onalespib. HT55 cells were treated with 25, 50, 75, 100, and 150 nM of onalespib, and LS174T cells were treated with 600, 800, 1,000, and 2,000 nM of onalespib. Equal amounts of DMSO in the highest concentrations were used as control. Cell lysates were prepared 24 h posttreatment. Cells were washed with 1× cold PBS followed by incubating with Pierce™ IP lysis buffer (Thermo Fisher Scientific, Sweden) containing Halt™ Protease and Phosphatase inhibitor cocktail (Thermo Fisher Scientific, Sweden) for 10 min on ice. The lysates were centrifuged at 15,000 rpm for 15 min at 4°C, and supernatants were collected and stored at -20°C. Protein concentration of lysates was assessed by Pierce BCA Protein Assay Kit (Thermo Scientific, Sweden). Samples were run on 10% Bis-Tris gels in 3-(N-morpholino)propanesulfonic acid (MOPS) running buffer (Novex™, NuPAGE^®^, Invitrogen, Thermo Fisher Scientific, Sweden). Thereafter, the separated proteins were transferred to a Polyvinylidene Difluoride (PVDF) membrane (Merck Millipore, Darmstadt, Germany) for 2 h with the constant voltage of 100 V at 4°C. The membranes were blocked in 5% bovine serum albumin (BSA) in PBS-Tween (0.1%) for 1 h and were then incubated with primary antibodies in 1% BSA in PBS-Tween (0.1%) at 4°C overnight. Primary antibodies targeted CEA (CD66e, 14-0669-82 Mouse monoclonal antibody, eBioscience, Invitrogen, Sweden), EGFR (ab52894, Rabbit monoclonal antibody, Abcam, UK), and AKT1,2,3 (ab179463, Rabbit monoclonal antibody, Abcam, UK). Glyceraldehyde-3-Phosphate Dehydrogenase (GAPDH) (ab8245, Mouse monoclonal antibody, Abcam, UK) or β-actin (ab8227, Rabbit polyclonal antibody, Abcam, UK) was used as a loading control. Membranes were washed and incubated with secondary antibodies (Invitrogen, Sweden) in PBS-Tween (0.1%) for 1 h on the following day. Thereafter, membranes were developed using SuperSignal™ West Pico PLUS Chemiluminescent Substrate (Thermo Scientific, Sweden) and Amersham™ Imagequant™ 800 (Thermo Fisher Scientific, Sweden). The experiments were repeated at least three times.

### XTT Cell Proliferation Assay

A defined number of cells were seeded in flat-bottom 96-well plates (8 × 10^3^ SNU1544 cells/well, 1 × 10^4^ HT55 cells/well, and 6 × 10^3^ LS174T cells/well) and incubated in an atmosphere containing 5% CO_2_ at 37°C for 48 h. For treatment, the medium was switched with onalespib-containing media (onalespib concentration range of 5–1×10^4^ nM) and incubated in an atmosphere containing 5% CO_2_ at 37°C for an additional 72 h. Equal amount of DMSO in the highest concentration was used as a DMSO control. The XTT cocktail (XTT activation reagent, XTT reagent, and the corresponding media) was prepared according to manufacturer’s instructions (ATCC protocol 30–1011 K, Manassas, VA, USA). Cells were incubated with XTT cocktail for 4 h in an atmosphere containing 5% CO_2_ at 37°C. The dual absorbance was then measured using a BioMark Microplate Reader (Bio-Rad Laboratories AB, Sweden). Significance was determined using one-way ANOVA followed by Tukey multiple comparison test.

### Binding Specificity Assay

In this study, 2.5 × 10^4^ cells were seeded in 48-well plates and incubated for 48 h at an atmosphere containing 5% CO_2_ at 37°C. After 24 h for HT55 cells, LS174T cells, and HT29 cells and 48 h for MKN45 and SNU1544 cells, 1 nM of ^177^Lu-DOTA-M5A with 100-fold blocking solution of unlabeled antibody (DOTA-M5A, 100 nM) was added to ≥3 wells each. After 24 h, the radioactive medium was removed, cells were washed 2–3 times with the basal corresponding media, and cells were trypsinized and counted using a cell counter (TC20™ Automated Cell Counter, BioRad, Sweden). Cell-associated radioactivity was counted in a gamma counter (1480 Wizard 3′, Wallace, Finland). Significance was determined using Student’s t-test.

### Real-Time Binding Measurement *via* Ligand Tracer

For binding measurements, 0.7 × 10^6^ MKN45 cells, 0.6 × 10^6^ SNU1544 cells, or 0.5 × 10^6^ LS174T cells were seeded in non-treated Petri dishes (Cat. No. 263991, Thermo Fisher Scientific) coated with polydopamine according to Ridgeview Instruments AB instructions. Polydopamine was used to enhance attachment of semi-adherent cells for real-time binding measurement experiments. In brief, dopamine hydrochloride (Sigma, Germany) dissolved in 10 mM Tris buffer (pH 8) to a final concentration of 2 mg/ml was added as droplets (500 μl) near the edge of dishes. The dishes were incubated for 3 h at room temperature before the remnant solution was removed, and dishes were rinsed with MQ water twice. The coated dishes were stored at 4°C until used. The abovementioned number of cells was suspended in 0.5 ml of the corresponding basal media and seeded on polydopamine followed by 1 h incubation at room temperature. Then, the basal medium was removed, and 10 ml of the corresponding complete medium was added to the dish. For HT55, 1.5 × 10^6^ cells were seeded on tilted cell culture-treated Nunc dishes (Cat. No. 150350, Thermo Fisher Scientific). All cells were incubated in an atmosphere containing 5% CO_2_ at 37°C. After 48 h incubation for MKN45 and SNU1544 cells and 24 h incubation for HT55 and LS174T cells, the real-time binding measurement was performed as previously described (53). In brief, the cell medium was switched to 3 ml of fresh medium prior to the start of the binding measurement. The binding of ^177^Lu-DOTA-M5A to cells was measured with LigandTracer Yellow (Ridgeview Instruments AB, Uppsala, Sweden) at room temperature. A baseline signal was collected for around 15 min before stepwise adding ^177^Lu-DOTA-M5A to final concentrations of 3 and 9 nM. Dissociation was initiated when the binding curve got sufficient curvature at least in one concentration by replacing the media with 3 ml fresh media. Polydopamine or plastic surface of an area with no cells on the same dish was used as the reference background area to investigate off-target binding of radiolabeled antibody. The reference background signal was automatically subtracted from the decay-corrected target area signal that resulted in a specific real-time binding curve of ^177^Lu-DOTA-M5A to the target cells. Kinetic interaction evaluation was performed with TraceDrawer 1.9.2 (Ridgeview Instruments AB, Uppsala, Sweden).

### Three-Dimensional Spheroid Models

The 96-well flat bottom plate (Sarstedt, Germany) was coated with 50 μl of 0.15% agarose (Sigma Aldrich, MO, USA) as previously described (54). Cells that form a 3D structure were then seeded (3,000 cells/well for HT55 and LS174T and 2,000 cells/well for SNU1544). MKN45 did not form a 3D structure and was therefore excluded for 3D model experiments. When the 3D structure (spheroids) reached 0.4 mm in diameter (3–4 days), they were treated with ^177^Lu-DOTA-M5A (5–80 kBq) and/or onalespib (25–1,000 nM). Onalespib concentrations and ^177^Lu-DOTA-M5A activities for the combination therapies were chosen based on the effect of monotreatments where either tumor growth or viability decreased significantly. DMSO, sodium/ammonium acetate buffer, ^177^Lu-DOTA, and unlabeled DOTA-M5A were chosen as control groups. After treatment, the spheroid growth was assessed using Canon EOS 700D photographing (Canon Inc., Japan) mounted on a Nikon Diaphot-TMD microscope (Nikon, Japan) every 3 days. Media were added once on the third day after treatment and exchanged once on the seventh day after treatment. Spheroids were followed individually for 12 days. The cross-section area and spheroid sizes were measured and analyzed using ImageJ 1.52p software (NIH, Bethesda, MD, USA) and normalized to the size at the start of treatment (size ratio). Significance was determined using one-way ANOVA followed by Tukey multiple comparison test.

### Viability of Three-Dimensional Spheroid Models

To assess the viability of the 3D models metabolically, the High-sensitivity (HS) alamarBlue^®^ assay (Invitrogen, MA, USA) was used (55) to evaluate the treatment effects. The viabilities of the HT55 and SNU1544 spheroid models were evaluated at three time points (Day 0; treatment start, Day 6; at the half-time of the treatment period and Day 12 at the assay endpoint). Due to the fact that the LS174T spheroid model behaved differently from the other models, the viability was assessed at five time points (Day 0, Day 3, Day 6, Day 9, and Day 12). In each well of the 96-well plate, 100 μl of medium was kept and 100 μl of 20% alamarBlue-containing media (v/v) were added in order to reach the final concentration of 10% (v/v). Plates were protected from light and incubated in an atmosphere containing 5% CO_2_ at 37°C (55). After 24 h of incubation, dual fluorescence absorbance was read using an ELISA reader (210 Infinite series, TECAN, Switzerland) at gain 90 constantly during the experiment. Significance was determined using one-way ANOVA followed by Tukey multiple comparison test.

### Spheroid Lysates and Western Blot Analyses

To assess the molecular mechanisms of onalespib and/or ^177^Lu-DOTA-M5A treatments and the combinations thereof, defined numbers of SNU1544 (1.5 × 10^5^/well) and LS174T cells (3.6 × 10^5^/well) were seeded on AggreWell™ 800 plates (Stemcell Technologies, Canada) according to the manufacturer’s instruction. Briefly, each well was covered with 500 µl of anti-adherent solution and centrifuged at 1,300×g for 5 min. Thereafter, each well was washed with 2 ml of basal media followed by adding 1 ml of complete media. Cells were harvested by trypsin, and a single-cell suspension was prepared. Cells were counted, and the aforementioned number of cells was then seeded with complete media to a total volume of 2 ml/well. The plate was immediately centrifuged at 100×g for 3 min to capture cells in the microwells. The plate was observed under microscope to verify that cells were distributed evenly among microwells. The plates were then incubated in an atmosphere containing 5% CO_2_ at 37°C for 4–5 days (pretreatment incubation). For treatment, 1 ml of media in each well were switched with media containing onalespib or ^177^Lu-DOTA-M5A and the combinations thereof. Spheroids were collected 24 h posttreatment, and lysates were prepared with Radioimmunoprecipitation Assay Buffer (RIPA) lysis buffer (Thermo Fisher Scientific, Sweden) supplemented with Halt™ Protease and Phosphatase inhibitor cocktail (Thermo Fisher Scientific, Sweden) followed by vortex/spin down cycles (30 s of vortexing followed by 30 s of spinning down, 30 s rest on ice) for 10–20 min. The lysates were centrifuged at 15,000 rpm for 15 min at 4°C, and supernatants were collected and stored at -20°C. Protein quantification of lysates was assessed by Pierce BCA Protein Assay Kit (Thermo Scientific, Sweden). The samples were separated on Sodium Dodecyl-Sulfate Polyacrylamide Gel Electrophoresis (SDS-PAGE), transferred to PVDF membrane, and blocked as described earlier. The membranes were incubated with primary antibodies targeting CEA (CD66e, 14-0669-82 Mouse monoclonal antibody, eBioscience, Invitrogen, Sweden), EGFR (ab52894, Rabbit monoclonal antibody, Abcam, UK), HSP90 (ab13492, Mouse monoclonal antibody, Abcam, UK), HSP70 (ADI-SPA-812, Rabbit polyclonal antibody, Enzo Life Science, USA), ERK1+ERK2 (ab115799, Rabbit polyclonal antibody, Abcam, UK), SMAD3 (51-1500, Rabbit polyclonal antibody, Invitrogen, Sweden), and Cleaved Poly [ADP-Ribose] Polymerase 1 (PARP1) (ab32064, Rabbit monoclonal antibody, Abcam, UK). GAPDH (ab8245, Mouse monoclonal antibody, Abcam, UK) was used as a loading control. The membranes were incubated with the secondary antibodies and developed as described previously.

### Synergy Calculations and Statistical Analysis

Synergy calculations and analyses were done using SynergyFinder 2.0 (56) on spheroid size ratios (12 days after treatment) and the spheroid viabilities at the experiment end point (12 days posttreatment) for the SNU1544 and HT55 spheroid models and at day 6 posttreatment for the LS174T spheroid model. Highest single agent (HSA) (57), Bliss (58), and Zero interaction potency (ZIP) (59) models were used in the current study for synergy calculation. These models are slightly different; Bliss and ZIP models are mostly used in drug combination effects and HSA is used for the maximal single-drug effect. Bliss model is suitable when two drugs act independently and ZIP suits when it is not expected that one drug potentiates the other one (60). All experiments included in this study were performed in a minimum of two independent replicates, and data are presented as pooled data from all replicates or from one representative experiment. Statistical data analysis for other experiments was performed using GraphPad Prism Version 9.1.0 (GraphPad Software Inc., La Jolla, CA, USA). Data are presented as the means ± standard deviation (SD) if not otherwise stated.

## Results

### ^177^Lu-Radiolabeling Characterization and Stability Assays

**Figure 1:**
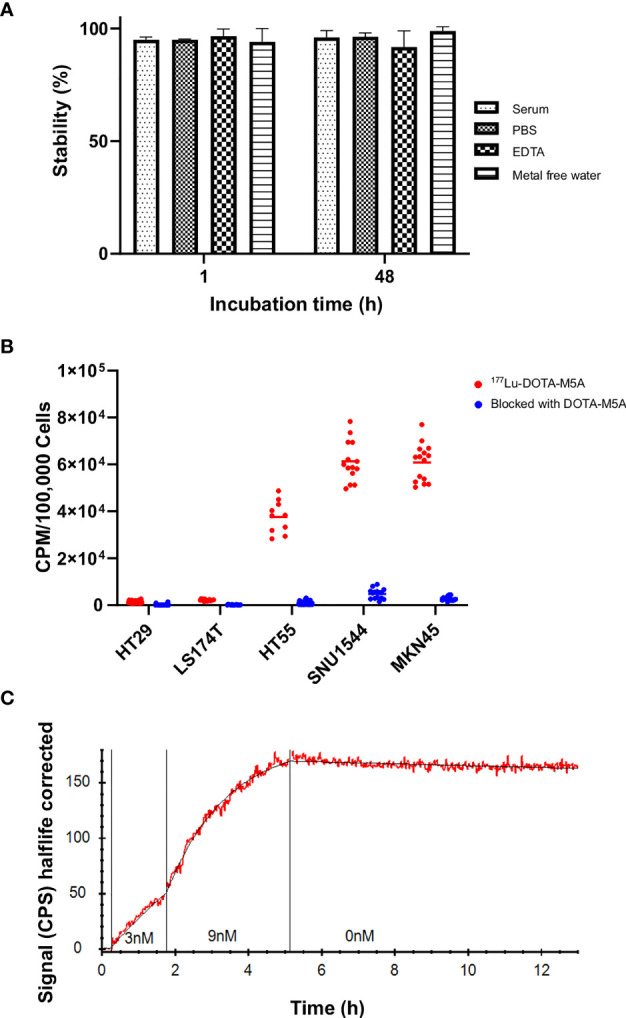
The ^177^Lu-DOTA-M5A stability, binding specificity assessment, and real-time binding measurement of ^177^Lu-DOTA-M5A to the target antigen on a panel of gastrointestinal cancer cell lines. **(A)** Stability assessment of ^177^Lu-DOTA-M5A at different conditions (PBS, mixture of mouse serum and PBS, EDTA condition, and metal-free water) 1 and 48 h after labeling. Graph displays stable ^177^Lu-DOTA-M5A (%) on the y axis and incubation time on the x axis. **(B)** Binding specificity of ^177^Lu-DOTA-M5A to the target antigen CEA on HT29, LS174T, HT55, SNU1544 (colorectal cancer cell lines) and MKN45 cell line (gastric cancer cell line). Graph displays signal [counts per minute (CPM)] per 100,000 cells on the y axis and tested cell lines on the x axis. **(C)** Representative binding curve of ^177^Lu-DOTA-M5A binding to MKN45 cells (CEA-positive gastric cancer cell line, red) as measured with LigandTracer Yellow. Black curve resulting from kinetic evaluation with a one-to-one binding model. Graph displays signal [counts per second (CPS)] half-life corrected on the y axis and time on the x axis. Binding curves for LS174T, HT55, and SNU1544 (CEA-positive colorectal cancer cell lines) are shown in **Supplementary Figure S5**. PBS, Phosphate-buffered saline; EDTA, Ethylenediamine tetraacetic acid.

The optimal ^177^Lu-labeling condition was determined for DOTA-M5A labeling (**Supplementary Figures S3** **,** **S4**). Both 1 and 2 h of incubation for labeling of DOTA-M5A (50 µg) with 15 MBq of ^177^Lu at 42°C resulted in more than 97% of radiolabeling yield (**Supplementary Figure S4**). The stability of ^177^Lu-DOTA-M5A was above 90% in all tested conditions (**Figure 1A**), indicating high stability and intact radioconjugation of ^177^Lu-DOTA-M5A under physiological conditions.

### ^177^Lu-DOTA-M5A Binds Specifically to Carcinoembryonic Antigen on Gastrointestinal Cancer Cell Lines

A binding specificity assay was first performed on a panel of GI cancer cell lines in order to assess the ^177^Lu-DOTA-M5A binding specificity and to validate the individual CEA levels. All cell lines demonstrated detectable binding of ^177^Lu-DOTA-M5A, blockable with unlabeled DOTA-M5A. This confirmed the antigen-specific binding of the conjugate and further supported that the radiolabeling did not interfere with antigen binding. Binding levels of unblocked ^177^Lu-DOTA-M5A were in line with literature data (61), with the MKN45 and SNU1544 cell lines demonstrating the highest signal, followed by HT55 and LS174T cells, and HT29 cells demonstrating the lowest signals (**Figure 1B**).

### Real-Time Binding Measurement

Real-time binding measurements of ^177^Lu-DOTA-M5A were performed on the cell line panel in order to assess the antibody–antigen interactions in terms of on-rate, off-rate, affinity, and type of interaction (**Figure 1C** and **Supplementary Figure S5**).

Measurements of ^177^Lu-DOTA-M5A on CEA-positive cells demonstrated high affinity binding and very slow dissociation (off-rate). Binding of ^177^Lu-DOTA-M5A to cell lines MKN45, SNU1544, HT55, and LS174T was well described by a one-to-one or Langmuir binding model. A representative example of ^177^Lu-DOTA-M5A interaction with CEA on MKN45 cells is shown in **Figure 1C**. Binding and interaction curves for SNU1544, HT55, and LS174T cell lines are shown in **Supplementary Figure S5**. The measured affinity of ^177^Lu-DOTA-M5A was quite similar in all cell lines, ranging between 11 and 94 pM (**Table 1**). This further validated the antigen-specific binding of ^177^Lu-DOTA-M5A demonstrated in the specificity assay (**Figure 1B**). The slow off-rate detected on CEA-positive cell lines indicated a stable interaction between ^177^Lu-DOTA-M5A and the cellular target. To assess the variation in kinetic parameters, for a typical cell line, the relative standard deviation for k_a_ was below 5%, while the k_d_ was less well-defined and could vary up to 40%.

**Table 1:** Binding characterization of ^177^Lu-DOTA-M5A interaction with CEA on MKN45, SNU1544, HT55, and LS174T cells as estimated from LigandTracer experiments by kinetic evaluation.

Cell line	k_a_ (× 10^4^ M^-1^ s^-1^)	k_d_ (× 10^-6^ s^-1^)	K_D_ (pM)
MKN45	1.9	1.4	74
SNU1544	0.9	0.9	94
HT55	1.5	0.9	63
LS174T	3.2	0.4	11

Summary of the association rate constant (k_a_), the dissociation rate constant (k_d_), and the equilibrium dissociation constant or affinity (K_D_), N = 2. Kinetic evaluation was performed with a two-step approach. First the k_d_ of the two replicates was estimated globally with the DissociationRate model. The acquired k_d_ was then used as a constant in a global one-to-one binding model to assess k_a_ and the affinity K_D_. pM, pico molar.

### Dose-Dependent and Antigen-Specific Therapeutic Effect of ^177^Lu-DOTA-M5A in Three-Dimensional Colorectal Spheroid Models

**Figure 2:**
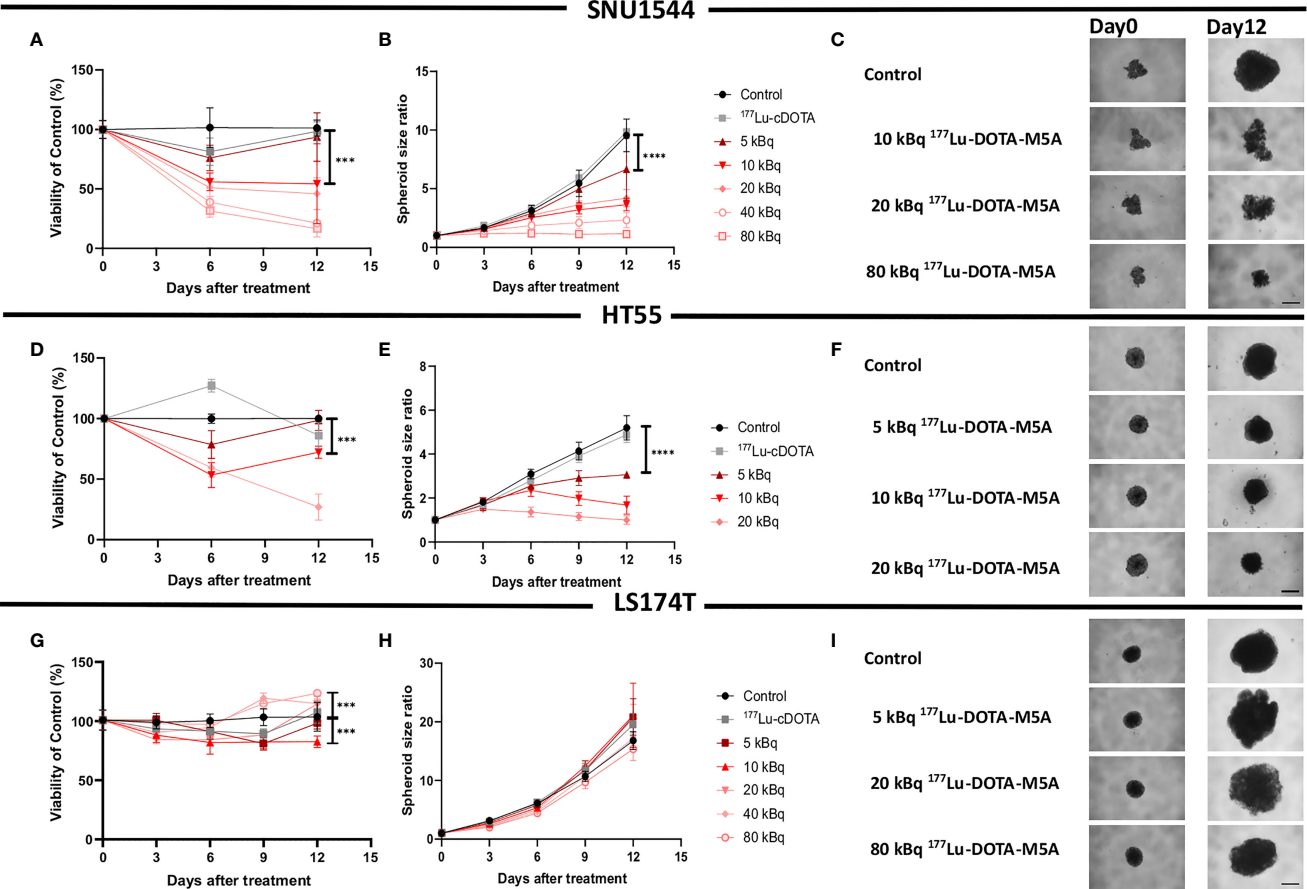
Monotherapy with ^177^Lu-DOTA-M5A in 3D spheroid models. Viability **(A)** and spheroid growth assessment **(B, C)** of the SNU1544 spheroid model treated with ^177^Lu-DOTA-M5A. Viability **(D)** and spheroid growth **(E, F)** assessment of the HT55 spheroid model treated with ^177^Lu-DOTA-M5A. Viability **(G)** and spheroid growth **(H, I)** assessment of the LS174T spheroid model treated with ^177^Lu-DOTA-M5A. The treatment activities are indicated in graphs and images. Graphs display either spheroid size ratio or viability (% of control) on the y axis and time after treatment on the x axis (mean ± standard deviation, n ≥ 4). *P ≤ 0.05, ***P ≤ 0.001, and ****P ≤ 0.0001. For treatments, the lowest concentration that caused a significant decrease in spheroid size/viability compared to untreated control was shown. Representative images of spheroids at first time point and at the endpoint are shown in panels **(C, F, I)**; scale bar: 400 µm.

The effects of ^177^Lu-DOTA-M5A as a monotherapy was then assessed in three 3D colorectal spheroid models, assessing both spheroid size over time and cellular viability (**Figure 2** and **Table 2**). In general, both spheroid size and viability measurements demonstrated antigen-specific and dose-dependent therapeutic effects of ^177^Lu-DOTA-M5A (**Figure 2** and **Table 2**).

**Table 2:** The spheroid size ratio and viability [mean ± standard deviation (SD)] of the SNU1544, HT55, and LS174T 3D spheroid models treated with monotreatment of either ^177^Lu-DOTA-M5A or onalespib at day 12 posttreatment (n ≥ 4).

Spheroid model	Treatment		Viability (% of control) ± SD	Spheroid size ratio ± SD
SNU1544 (high CEA expression)	Untreated control	0	100	9.5 ± 1.3
	^177^Lu-DOTA-M5A (kBq)	10	54 ± 13	3.6 ± 0.5
		20	46 ± 6	4.1 ± 0.7
		40	21 ± 1	2.3 ± 0.6
	Onalespib (nM)	250	68 ± 15	6.8 ± 1.1
		350	66 ± 27	4.6 ± 0.5
		450	39 ± 17	2.6 ± 0.2
HT55 (high CEA expression)	Untreated control	0	100	5.2 ± 0.5
	^177^Lu-DOTA-M5A (kBq)	5	94 ± 20	3 ± 0.1
		10	54 ± 30	1.6 ± 0.4
		20	27 ± 10	1 ± 0.2
	Onalespib (nM)	25	75 ± 11	5 ± 0.6
		50	69 ± 5	3.5 ± 0.3
		75	33 ± 6	2.5 ± 0.3
LS174T (low CEA expression)	Untreated control	0	100	16.8 ± 1.5
	^177^Lu-DOTA-M5A (kBq)	10	82 ± 5	21 ± 5.5
		40	114 ± 3	17.2 ± 0.4
		80	123 ± 2	15.3 ± 2
	Onalespib (nM)	600	90 ± 3	12.4 ± 1
		800	82 ± 4	4.4 ± 0.4
		1,000	55 ± 2	1.6 ± 0.1

SD, standard deviation.

In the high CEA-expressing model SNU1544, treatment with, e.g., 10 and 40 kBq ^177^Lu-DOTA-M5A reduced spheroid cell viability to 54% ± 13% and 21% ± 1%, respectively, compared to untreated controls (100%) at the assay endpoint (day 12), with the corresponding spheroid size ratios of 3.6 ± 0.5 and 2.3 ± 0.6 compared to 9.5 ± 1.3 for the untreated controls at the same time point (**Figures 2A–C** and **Table 2**). Likewise, in the high CEA-expressing HT55 model, the clear dose-dependent effects of ^177^Lu-DOTA-M5A reflected on both spheroid size and viability (**Figures 2D–F** and **Table 2**). At day 12, 10 and 20 kBq ^177^Lu-DOTA-M5A reduced spheroid cell viability to 72% ± 5% and 27% ± 10%, respectively, compared to untreated controls (100%) at the assay endpoint (day 12), and the corresponding spheroid size ratios were 1.6 ± 0.4 and 1 ± 0.2 compared to 5.2 ± 0.5 for the untreated control. In the low CEA-expressing model LS174T, no significant reducing effects of ^177^Lu-DOTA-M5A treatment were observed in spheroid size evaluation. At treatment with lower activities of ^177^Lu-DOTA-M5A, a trend of reduced spheroid density could be noted at the experimental endpoint, reflected in increased spheroid diameter but not increased viability. Viability assessments demonstrated minor changes over time in the treatment groups, with some marked changes for 10 and 80 kBq ^177^Lu-DOTA-M5A at the end point (**Figures 2G–I** and **Table 2**). None of the control groups (sodium acetate, ^177^Lu-DOTA, and unlabeled DOTA-M5A) significantly impacted the spheroid size and viability (**Supplementary Figure S6**).

### Effects of Onalespib Monotherapy in Colorectal Cancer Models

In order to assess the possibility to potentiate the effect of ^177^Lu-DOTA-M5A with the HSP90 inhibitor onalespib, the effect of onalespib monotherapy on CEA expression levels was first evaluated. Western blot analysis displayed that CEA expression was not affected by onalespib treatment in any of the assessed colorectal cancer cell lines, while known HSP90-related markers, such as EGFR and AKT1,2,3, displayed alterations. Moreover, the assessed 3D spheroid lysates of SNU1544 and LS174T displayed higher CEA expression compared to their 2D lysates (**Supplementary Figure S7**).

**Figure 3:**
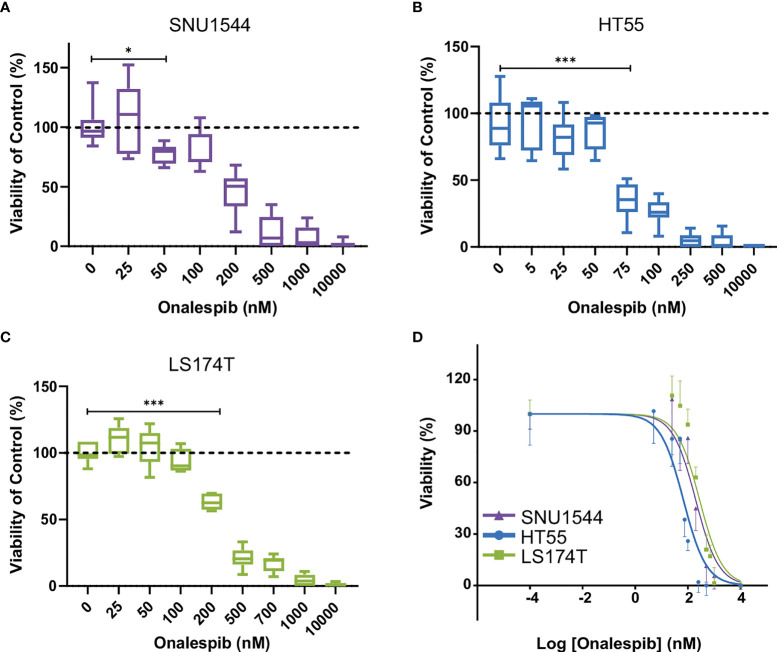
Characterization of colorectal cancer cell lines. Effect of onalespib treatments on viability of SNU1544 cells **(A)**, HT55 cells **(B)**, and LS174T cells **(C)**. Graphs display viability (% of control) on the y axis and onalespib concentration (nM) on the x axis. For treatments, the lowest concentration that caused a significant decrease of viability compared to the untreated control was shown. *P ≤ 0.05 and ***P ≤ 0.001. **(D)** XTT proliferation assay of 2D cultures. Graph displays viability (%) on the y axis [mean and 95% confidence interval (CI), n ≥ 4] and logarithmic concentrations of onalespib on the x axis. CI, confidence interval.

Furthermore, to evaluate the half-maximal inhibitory concentration (IC50), the growth-inhibitory effect of onalespib was assessed by XTT cell proliferation assays on three different colorectal cancer cell lines. All cell lines responded to onalespib monotherapy, albeit with different sensitivities (**Figure 3**). HT55 cells were the most sensitive cells with IC50 = 67 nM (95% CI, 50–88 nM). SNU1544 cells were less sensitive with IC50 = 197 nM (95% CI, 148–260 nM), and LS174T was the least sensitive cell line with IC50 = 362 nM (95% CI, 262–498 nM).

**Figure 4:**
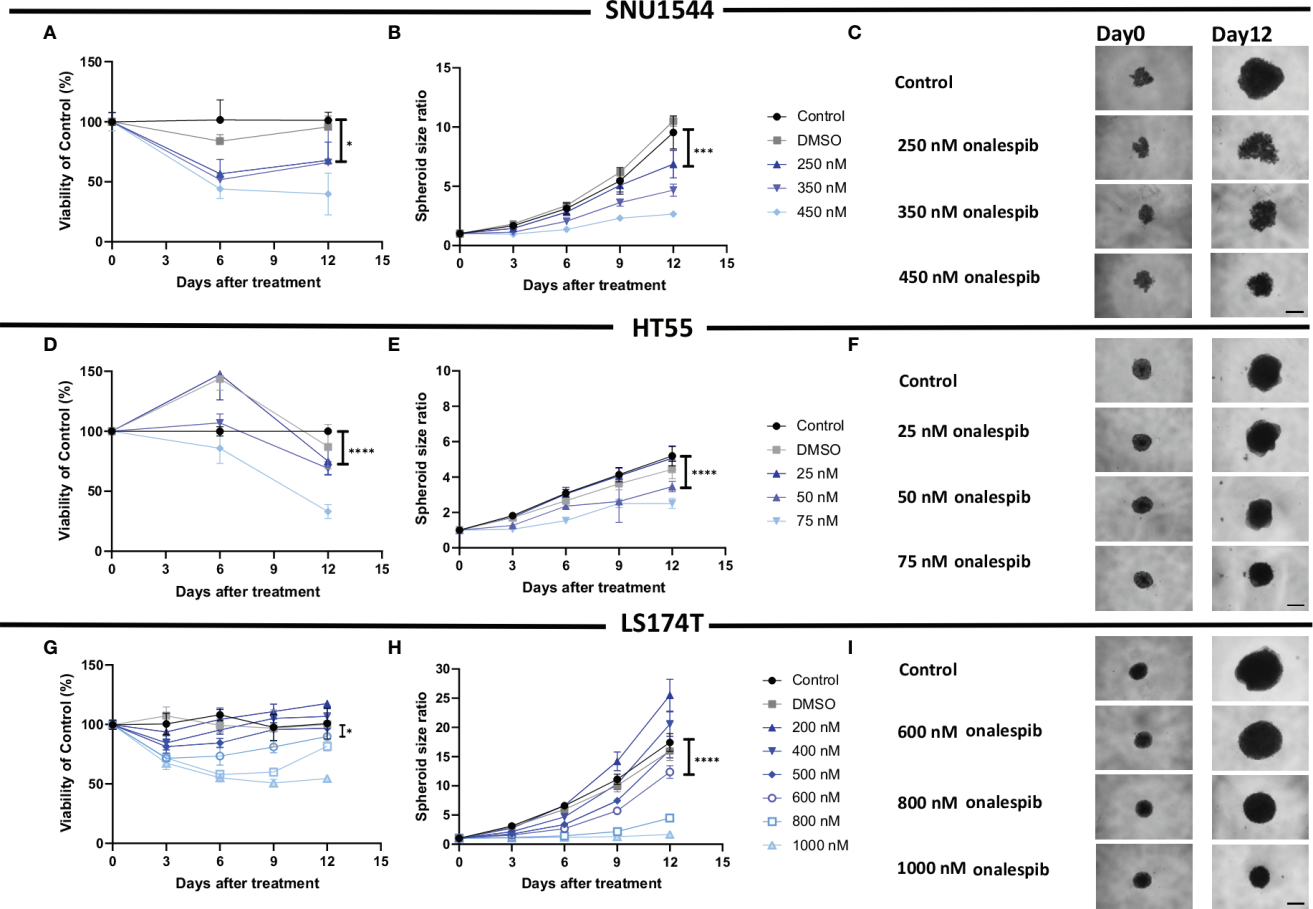
Monotherapy with onalespib in 3D spheroid models. Viability **(A)** and spheroid growth assessment **(B)** of the SNU1544 spheroid model treated with onalespib. Viability **(D)** and spheroid growth **(E)** assessment of the HT55 spheroid model treated with onalespib. Viability **(G)** and spheroid growth **(H)** assessment of the LS174T spheroid model treated with onalespib. The treatment concentrations are indicated in the graphs and images. Graphs display either spheroid size ratio or viability (% of control) on the y axis and time after treatment on the x axis (mean ± standard deviation, n ≥ 4). For treatments, the lowest concentration that caused a significant decrease in spheroid size/viability compared to the untreated control was shown. *P ≤ 0.05, ***P ≤ 0.001, and ****P ≤ 0.0001. Representative images of spheroids at first time point and at the endpoint are shown in panels **(C, F, I)**; scale bar: 400 µm.

Inhibitory growth effects of onalespib as a single drug were then assessed in 3D spheroid models by both spheroid size and viability measurements, demonstrating that all three assessed cell lines were sensitive to onalespib also in a 3D setting. Onalespib concentrations above 250 nM caused marked reduction of SNU1544 spheroid size and viability (**Figures 4A–C** and **Table 2**). Treatment with 250 and 450 nM of onalespib resulted in viability reduction to 68% ± 7% and 39% ± 7% of untreated controls (100%), respectively, at the assay endpoint, and the corresponding spheroid size ratios were 4.6 ± 0.5 and 2.6 ± 0.2 compared to 9.5 ± 1.3 for the untreated controls. The HT55 spheroid model demonstrated a higher sensitivity to onalespib compared to the SNU1544 model, with concentrations above 50 nM leading to significantly smaller spheroids and demonstrating markedly reduced viability at 25 nM at the assay endpoint (**Figures 4D–F** and **Table 2**). The LS174T spheroid model demonstrated the lowest sensitivity to onalespib, with at least 600 nM required to achieve significant therapeutic effects (**Figures 4G–I** and **Table 2**). Moreover, at 200–400 nM, a trend of increased spheroid diameter could be noticed at the experimental endpoint, likely reflecting reduced spheroid density.

The dose-dependent effects of onalespib on the SNU1544 spheroid model were clearly observed at early time points and persisted until the end of the experiment. The HT55 spheroid model demonstrated therapeutic effects mainly at later time points. In the LS174T spheroid model, the therapeutic effects were apparent at day 6 posttreatment, but only the highest concentration of onalespib was able to cause a lasting viability reduction and unchanged spheroid size until the end of experiments. DMSO did not demonstrate any marked effect in any of the experiments (**Supplementary Figure S6**).

Thus, both 2D and 3D experiments demonstrated that all three cell lines were sensitive to onalespib treatment, and the 3D spheroid model results were in line with 2D data regarding sensitivity levels, identifying HT55 as the most sensitive cell line and LS174T as the least sensitive to onalespib treatment.

### Combination Therapy of ^177^Lu-DOTA-M5A and Onalespib Potentiates Therapeutic Effects in Three-Dimensional Colorectal Cancer Spheroids

**Figure 5:**
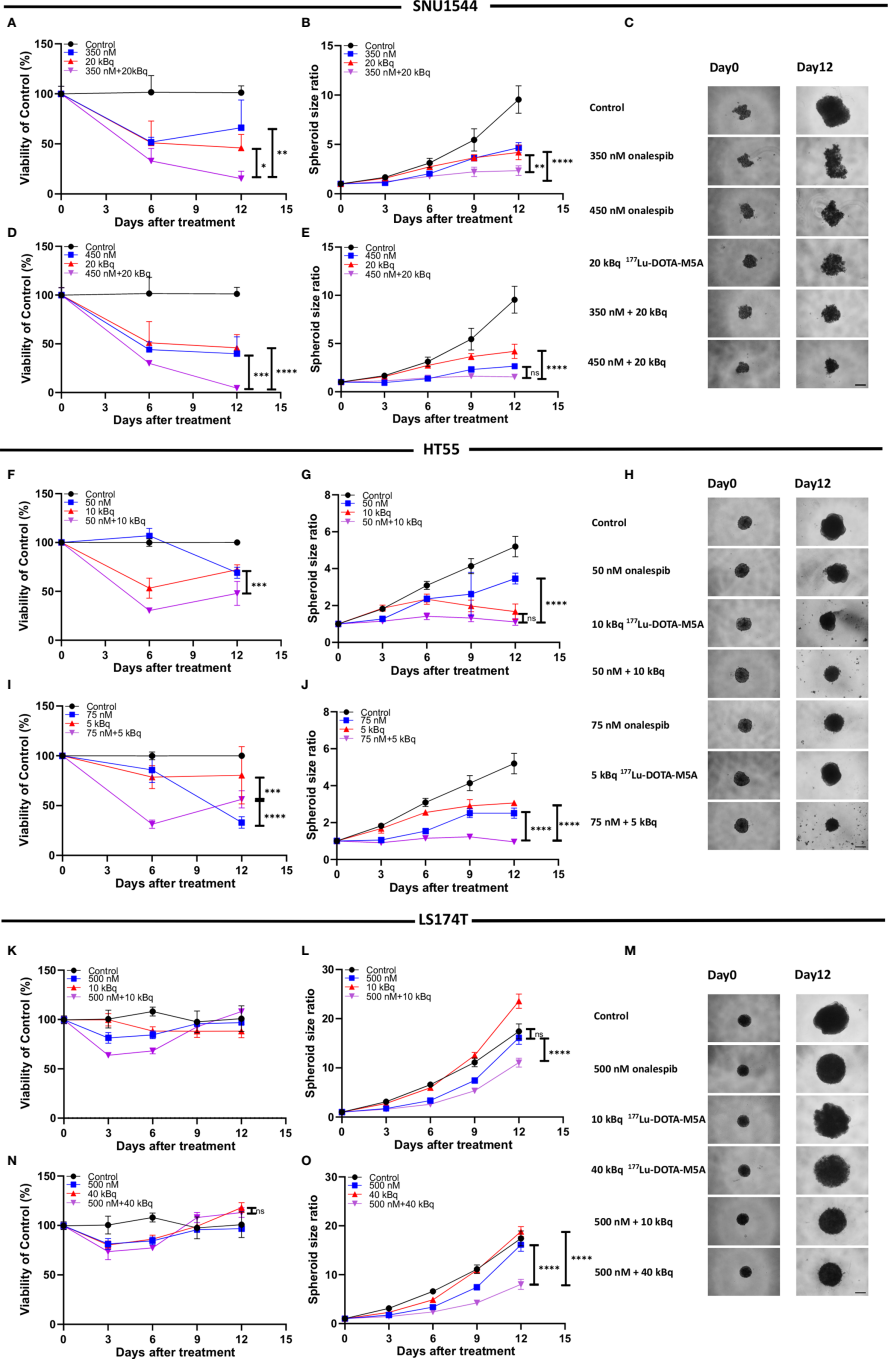
Combination therapy with ^177^Lu-DOTA-M5A and onalespib in 3D spheroid model SNU1544 **(A–E)**, HT55 **(F–J)**, and LS174T **(K–O)**. Treatments with ^177^Lu-DOTA-M5A, onalespib, and combination treatments compared to the corresponding monotreatments. Graphs display either spheroid size ratio or viability (% of control) on the y axis (means ± standard deviation, n ≥ 4) and time after treatment on the x axis. ***P ≤ 0.001 and ****P ≤ 0.0001 and ns, not significant. Representative images of spheroids at first time point and endpoint are shown in panels **(C, H, M)**; scale bar: 400 µm. *P ≤ 0.05 and **P ≤ 0.01.

**Figure 6:**
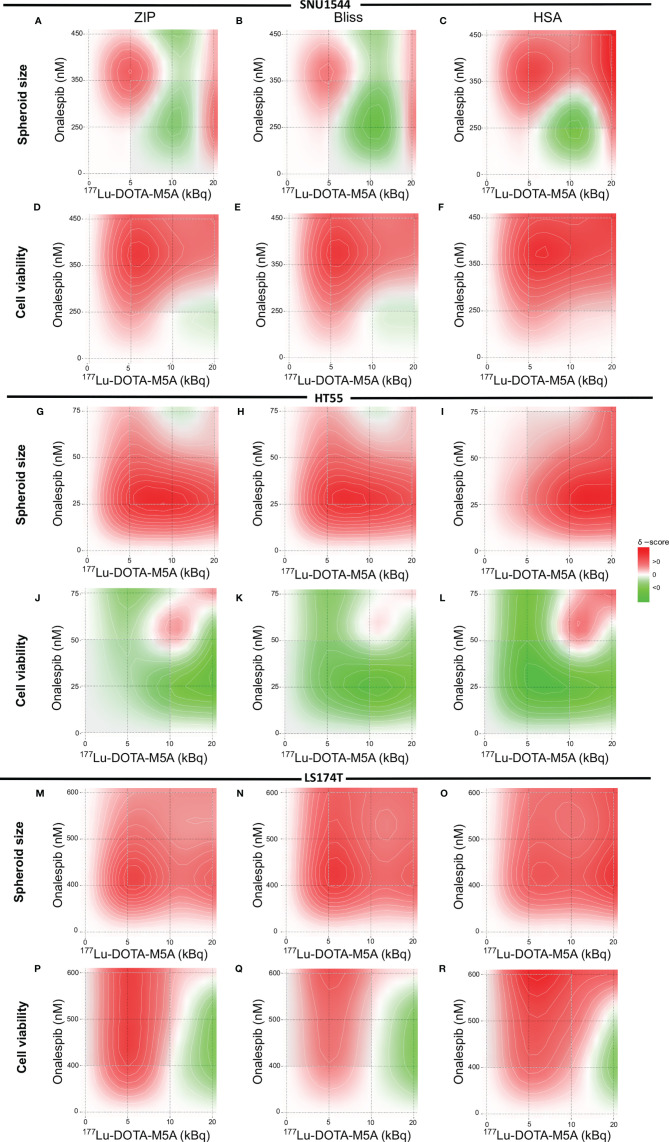
Heat map of synergistic effects. The SNU1544 spheroid size ratio **(A–C)** and viability **(D–F)**, the HT55 spheroid size ratio **(G–I)** and viability **(J–L)** at day 12 posttreatment, and the LS174T spheroid size ratio **(M–O)** and viability **(P–R)** at day 6 posttreatment. Graphs display onalespib concentration (nM) on the y axis and activity of ^177^Lu-DOTA-M5A (kBq) on the x axis. Values equal to zero (white area) are counted as additive effect. Values above zero (white to red area) are counted as synergistic effect, and values below zero (white to green area) are counted as antagonistic effect.

While both ^177^Lu-DOTA-M5A and onalespib demonstrated therapeutic effects as monotherapies, the combination therapies demonstrated the strongest therapeutic effects for numerous combinations. Two selected combinations for each spheroid model are shown in **Figure 5** (the rest are shown in **Supplementary Figures S8** **–** **S10**). All assessed combinations are summarized in **Table 3** and accounted for the synergy calculations illustrated in **Figure 6**. In the high CEA-expressing models SNU1544 and HT55, both viability and spheroid growth in the combination treatments were reduced to lower levels than the monotreatments, with clear combination benefits visible already at study midway for the selected combinations (**Figures 5A–J**).

**Table 3:** The spheroid size and viability [mean ± standard error of mean (SEM)] of the SNU1544 and HT55 spheroid models treated with the combination treatment of ^177^Lu-DOTA-M5A and onalespib at day 12 posttreatment (n ≥ 4).

Spheroid model	Treatment		Viability (% of control) ± SEM		Spheroid size ratio ± SEM	
				Day 12		Day 12
SNU1544 (high CEA expression)	^177^Lu-DOTA-M5A (kBq) + Onalespib (nM)	250 + 5		44 ± 7		5.6 ± 0.7
		250 + 10		58 ± 10		5.4 ± 0.2
		250 + 20		42 ± 9		2.9 ± 0.3
		350 + 5		18 ± 3		3 ± 0.1
		350 + 10		17 ± 1		2.3 ± 0.2
		350 + 20		15 ± 3		2.3 ± 0.1
		450 + 5		11 ± 1		2.3 ± 0.2
		450 + 10		11 ± 1		2.3 ± 0.2
		450 + 20		5 ± 0.2		1.5 ± 0.1
HT55 (high CEA expression)	^177^Lu-DOTA-M5A (kBq) + Onalespib (nM)	25 + 5		109		2.5 ± 0.1
		25 + 10		104 ± 3		1.4 ± 0.2
		25 + 20		48 ± 4		1
		50 + 5		95 ± 5		1.5 ± 0.1
		50 + 10		48 ± 5		1.1 ± 0.1
		50 + 20		37 ± 2		0.8
		75 + 5		56 ± 4		0.9 ± 0.1
		75 + 10		30 ± 3		1.1
		75 + 20		19 ± 1		1 ± 0.1
			**Day 6**	**Day 12**	**Day 6**	**Day 12**
LS174T (low CEA expression)	^177^Lu-DOTA-M5A (kBq) + Onalespib (nM)	400 + 10	78 ± 1	106 ± 2	3.6 ± 0.1	16.3 ± 0.3
		400 + 20	81 ± 2	125 ± 2	3.8 ± 0.1	16.4 ± 0.1
		400 + 40	84 ± 1	131 ± 2	3.3	13.1 ± 0.2
		500 + 10	68 ± 1	108 ± 1	2.6 ± 0.1	11.1 ± 0.4
		500 + 20	73 ± 2	121 ± 1	2.8 ± 0.1	11.6 ± 0.3
		500 + 40	77 ± 1	113 ± 2	2.4	8 ± 0.4
		600 + 10	68 ± 2	109 ± 2	2 ± 0.1	7.3 ± 0.2
		600 + 20	73 ± 2	110 ± 1	2 ± 0.1	6.8 ± 0.2
		600 + 40	72 ± 2	113 ± 2	2	6.6 ± 0.3

The spheroid size and viability [mean ± standard error of mean (SEM)] of the LS174T spheroid model treated with the combination treatment of ^177^Lu-DOTA-M5A and onalespib at both days 6 and 12 posttreatment (n ≥ 4). Monotreatment and untreated control data are summarized in **Table 2**. SEM, standard error of mean.

In the LS174T spheroid model, the least sensitive to onalespib therapy and with the lowest CEA expression, the combination treatments clearly inhibited spheroid growth more efficiently compared to monotreatments already at day 6 posttreatment, lasting until the end of the experiments. The viability also decreased until day 6 posttreatment; however, the cells recovered at later time points (**Figures 5K–O**).

Consequently, the growth inhibition results demonstrated that the combination treatments could mediate stronger therapeutic effects compared to monotreatments in all three spheroid models, also supported by the viability assays. Negative control groups did not display any significant alteration in spheroid size and viability (**Supplementary Figure S6**).

Synergistic effects were assessed with ZIP, Bliss, and HSA synergy models and were in line with spheroid growth and viability results (**Figure 6**). Synergistic evaluation on SNU1544 spheroid size ratios demonstrated that both the lowest (5 kBq) and the highest (20 kBq) assessed amounts of ^177^Lu-DOTA-M5A combined with onalespib resulted in synergistic effects (**Figures 6A–C**). Synergy calculations of SNU1544 viability illustrated that at least seven out of nine assessed combinations displayed synergistic effects (**Figures 6D–F**). In the HT55 spheroid model, the combinations of all ^177^Lu-DOTA-M5A activities with 25 and 50 nM of onalespib resulted in synergistic effects when spheroid size ratios were assessed (**Figures 6G–I**), and three of these also demonstrated synergistic effects in viability reduction (**Figures 6J–L**). In the LS174T spheroid model, synergistic effects were demonstrated in all nine combinations at day 6 posttreatment assessing spheroid size ratios (**Figures 6M–O**) and for viability in seven of the combinations (**Figures 6P–R**).

Overall, the synergistic evaluations indicated that for high CEA-expressing tumor models, such as the SNU1544 and HT55, combining 177Lu-DOTA-M5A and onalespib eventually lead to synergistic therapeutic effects in some combinations regarding both spheroid size ratio and viability at the end of the experiments. In the fast-growing tumor model LS174T, synergistic effects were mainly demonstrated at earlier time points regarding both spheroid size ratio and viability. The synergy scores are summarized in **Table 4**.

**Table 4:** The synergy scores of ZIP, Bliss, and HSA synergy models on both viability and spheroid size ratio of the SNU1544 and HT55 spheroid models (at day 12 posttreatment) and the LS174T spheroid model (at day 6 posttreatment).

3D model	Evaluation Day	Feature	Synergy model		
			ZIP	Bliss	HSA
SNU1544	12	Viability	19.2	19.2	27.8
		Spheroid size	1.4	-0.1	8.9
HT55	12	Viability	-6.7	-19.8	-17.1
		Spheroid size	11.6	11.7	15.8
LS174T	6	Viability	3.7	2.4	6.5
		Spheroid size	15.9	15.2	15.8

Positive values indicate a synergistic effect, and negative values indicate an antagonistic effect.

### Combination Therapy of ^177^Lu-DOTA-M5A and Onalespib Downregulates HSP90 Client Proteins and Potential Carcinoembryonic Antigen-Involved Pathways

**Figure 7:**
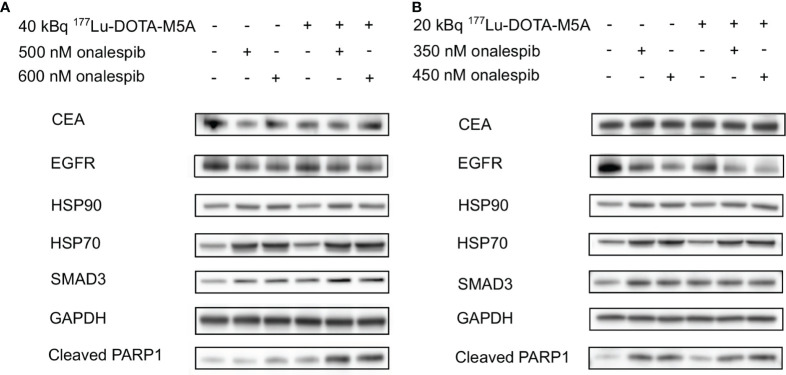
Western blot analysis of the treated 3D spheroid models. Western blot analysis of the LS174T-treated spheroids **(A)** and the SNU1544-treated spheroids **(B)** targeting CEA, EGFR, HSP90, HSP70, SMAD3, and Cleaved PARP1 24 h after treatment. GAPDH was used as a loading control. Representative blots are shown. CEA, carcinoembryonic antigen; EGFR, epidermal growth factor receptor; HSP90, heat shock protein 90; HSP70, heat shock protein 70; Cleaved PARP1, poly adenosine diphosphate-ribose polymerase; GAPDH, glyceraldehyde 3-phosphate dehydrogenase.

HSP90 client proteins and downstream signaling markers, a related co-chaperone, as well as potentially relevant markers to CEA, and an apoptosis marker, were assessed in the present study on lysates of 3D spheroids (SNU1544 and LS174T) 24 h after treatment with ^177^Lu-DOTA-M5A or onalespib and the combination thereof (**Figure 7**). No marked differences of CEA or HSP90 expression levels were observed in the treatment groups compared to the control group regardless of cell line. While EGFR was downregulated in the onalespib monotreatment group in a dose-dependent manner in both cell lines, effects were even stronger in the combination group for the SNU1544 cell line. Assessing HSP70, a related co-chaperone to HSP90 revealed that ^177^Lu-DOTA-M5A treatment did not influence HSP70 expression compared to the control; however, both onalespib and the combination treatments induced HSP70 upregulation in both assessed cell lines. Moreover, since other studies previously demonstrated that CEA interacts with the TGF-β receptor and induces proliferation in colorectal cancer cells (16, 62), SMAD family member 3 (SMAD3) expression was evaluated as a downstream marker of the TGF-β receptor. For both cell lines, SMAD3 was upregulated in all treatment groups compared to the control. To investigate whether the ^177^Lu-DOTA-M5A and/or onalespib treatment induced apoptosis, cleaved PARP1 expression was examined. In line with the viability assessment, marked cleaved PARP1 upregulation in the combination treatments compared to monotreatments and control was displayed in the LS174T spheroid model. The SNU1544 spheroid model displayed cleaved PARP1 upregulation in all treatment groups.

## Discussion

RIT is a promising approach for cancer therapy. For colorectal cancer, CEA has been demonstrated to be a suitable cancer-associated target for this application (7, 11). CEA targeting using hT84.66 M5A mAb has previously been assessed for RIT potential using ^90^Y as a therapeutic radionuclide, with promising results in a Phase I clinical study (11). However, labeling with ^177^Lu, a well-established and suitable radionuclide for cancer therapy, has not previously been assessed for RIT with an anti-CEA human mAb as a potential candidate for colorectal cancer therapy.

Although promising, there are however also potential limitations of RIT using full-sized antibodies, including restricted tumor penetration and dose-limiting bone marrow exposure (63). These limitations have encouraged exploration of combination therapies to potentiate therapeutic effects, including combining CEA-targeted therapy with chemotherapy drugs or immunocytokines with promising results (6, 8, 12, 29, 64). HSP90 inhibitors have previously demonstrated preclinical and clinical potential both as a single drug and in combination with external radiation and/or chemotherapy for GI tumors (65–69). The HSP90 inhibitor onalespib was recently demonstrated to have radiosensitizing properties when combined with either external beam radiation (45) or molecular radiotherapy through ^177^Lu-DOTATATE in tumor xenografts (70).

In the present study, the anti-CEA M5A mAb was successfully radiolabeled with ^177^Lu for the first time and combined with the HSP90 inhibitor onalespib. Both 2D cell experiments and 3D multicellular spheroid models were then used to further characterize the binding of ^177^Lu-DOTA-M5A to cells and to assess potential therapeutic effects for both monotherapies and combination approaches.

The radiolabeling of the DOTA-M5A antibody was first optimized and characterized for labeling yield, purity, and stability. Results demonstrated high yields and purity, and stability was retained for at least 48 h post-radiolabeling (**Figure 1A**). Real-time binding measurements on a panel of cell lines demonstrated picomolar binding affinity of the conjugate, with retained binding over 12 h (**Figure 1C** and **Supplementary Figure S5**). This indicated stable binding of ^177^Lu-DOTA-M5A on all tested CEA-positive cell lines with a very slow off-rate. The one-to-one interaction model described the ^177^Lu-DOTA-M5A and CEA interaction well. In specificity assays, the binding of ^177^Lu-DOTA-M5A could be blocked by an excess of unlabeled DOTA-M5A on all CEA-positive cells, demonstrating specific binding of the conjugate (**Figure 1B**). The binding levels reflected the antigen density of the cells and were in line with previous data (61). These results demonstrate that DOTA-M5A can be successfully labeled with ^177^Lu with retained antigen binding and also validated the CEA expression on assessed cell lines.

The potential therapeutic effects of ^177^Lu-DOTA-M5A were then assessed in 3D spheroid models with varying CEA expressions. Results demonstrated CEA-specific and dose-dependent effects of the conjugate (**Figure 2**). Monotherapy with ^177^Lu-DOTA-M5A reduced both spheroid size and viability in the high CEA-expressing SNU1544 and HT55 spheroid models, whereas the low CEA-expressing LS174T spheroid model did not display any significant spheroid size or viability reduction at the study endpoint. These results demonstrate that ^177^Lu-DOTA-M5A mediates dose-dependent and CEA-specific therapeutic effects in CEA-positive colorectal cancer models and may potentially lead to a promising conjugate to pursue for RIT of CEA-positive colorectal cancer. Moreover, the results emphasize the need to potentiate RIT effects in order to reach also the tumors with a lower antigen expression.

The growth-inhibitory effects of the HSP90 inhibitor onalespib were then assessed on the CEA-positive colorectal cancer cell lines (SNU1544, HT55, and LS174T), as they were considered most relevant for subsequent combination studies. Effects of onalespib treatment were studied in both monolayer cultures with XTT proliferation assays, as well as in 3D spheroid models by longitudinal spheroid growth measurements and cell viability assays (**Figures 3**, **4**). Onalespib inhibited all assessed cell lines, albeit in varying degrees. In monolayer viability assays, the HT55 was the most sensitive cell line with IC50 of 66.9 nM (95% CI, 50–88 nM), and LS174T was the least sensitive one with IC50 of 361.7 nM (95% CI, 262–498 nM) (**Figure 3**). These results were further validated in 3D spheroid models, which demonstrated that onalespib mediated dose-dependent growth inhibition and reduced cell viability, with the highest observed sensitivity for HT55 spheroid model followed by SNU1544 and LS174T spheroid models, demonstrating the lowest sensitivity. These results support HSP90 inhibition as a potential therapy for colorectal cancer, in line with recent clinical studies where inhibitors such as onalespib have demonstrated efficacy in solid tumors including colorectal cancers alone and in combination with other therapies (43, 71).

Interestingly, higher doses of onalespib were required to achieve therapeutic effects in the 3D spheroid models compared to the monolayer XTT assays. This is in agreement with previous studies comparing the effects of anticancer drugs in monolayer and 3D spheroid models, in which the latter illustrated more similarity to *in vivo* models in terms of drug penetration, cell-to-cell communication, and simulating essential tumor microenvironment factors, such as oxygen and nutrient gradients (72, 73). This emphasizes the important role of 3D models in the assessment of anticancer drugs. Moreover, the large span in onalespib sensitivity in the spheroid models also illustrates the heterogeneity within colorectal cancers and the need for combination treatments in order to widen the therapeutic window.

In the present work, ^177^Lu-DOTA-M5A RIT was combined with onalespib to assess potential therapeutic effects on spheroid growth and viability (**Figures 5**, **6**). As expected, the high CEA-expressing and onalespib-sensitive models SNU1544 and HT55 demonstrated clear combination effects of ^177^Lu-DOTA-M5A with onalespib, where both viability and spheroid growth were reduced to a higher extent than those with the monotherapies. For the SNU1544 spheroid model, the effect of 450 nM of onalespib on spheroid sizes was too strong to clearly demonstrate any combination benefits (**Figure 5E**), while the viability assessment displayed benefit of the combination treatment with significantly less cell viability (**Figure 5D**). Moreover, for the HT55 model, the effect of 10 kBq ^177^Lu-DOTA-M5A was too strong to clearly demonstrate any combination benefits on spheroid sizes (**Figure 5G**), whereas the viability assessment illustrated that the combination treatment resulted in lower spheroid viability compared to the corresponding monotreatments (**Figure 5F**). These results suggest a need for evaluating 3D spheroid models both by spheroid sizes and viability to gain a better grasp of the therapeutic effects. These 3D spheroid model experiments as a whole demonstrated that the therapeutic effects of combination treatment were mediated by both cellular CEA expression levels and the sensitivity to onalespib.

Surprisingly, clear combination effects were also found for the low CEA-expressing and the least onalespib-sensitive spheroid model LS174T. It should be noted that an increase of CEA expression was observed for the LS174T 3D spheroids compared to 2D models, in line with previous observations (74). Nevertheless, whereas 10 kBq of ^177^Lu-DOTA-M5A mediated no significant therapeutic effects as a monotherapy in this model, a combination of 10 kBq ^177^Lu-DOTA-M5A with 500 nM of onalespib resulted in reduced viability up to 6 days posttreatment (**Figure 5K**), followed by clear spheroid growth inhibition until the study endpoint at day 12 (**Figure 5L**). Consequently, the results indicated that combination therapy with ^177^Lu-DOTA-M5A and onalespib could potentiate the therapeutic effects in all studied 3D models at selected doses, albeit to different degrees. This reflects the potential of combination treatments as a way to overcome treatment resistance and widen the patient population eligible for RIT. Synergy calculations (**Figure 6**) further validated the effects of the combination treatments, although with varying optimal dose combinations for the different 3D spheroid models, reflecting the heterogeneity of colorectal cancers and the need to further individualize patient treatments.

To further evaluate the mechanisms and molecular effects of selected combination treatments, western blot analysis was performed on 3D tumor lysates 24 h posttreatment (**Figure 7**). The results validated previously known onalespib-mediated effects, such as downregulation of EGFR and upregulation of HSP70 (70, 71), demonstrating clear effects of HSP90 inhibition on a molecular level. Interestingly, in the high CEA-expressing spheroid model SNU1544, a combination with ^177^Lu-DOTA-M5A reduced EGFR levels even more than onalespib alone, indicating an even more efficient block of the EGFR pathway in this group, which is in line with a previous study (46).

The role of the TGF-β family proteins in carcinogenesis is complicated; however, it was previously found that CEA expression is closely correlated with the TGF-β pathway intermediate proteins such as SMAD3 (75). It has previously been shown that there is cross talk between CEA and the TGF-β pathway, where CEA targeting restored the TGF-β signaling and its ability to inhibit proliferation in colorectal cancer cells (16, 76). Consequently, SMAD3 expression was evaluated as a downstream marker of the TGF-β receptor in the present study. Our results demonstrated SMAD3 upregulation in all treatment groups, indicating a stimulation of the TGF-β pathway. Previous reports have indicated that both onalespib treatment and RIT can mediate apoptosis in cancer cells (42, 46, 77). In the present study, cleaved PARP1 was used as an apoptosis marker. PARP1 plays a critical role in DNA repair and genome stability and is cleaved in apoptosis (mainly by caspases 3 and 7), forming two fragments of 24 and 89 kDa (78–81). Results demonstrated a clear and dose-dependent increase in all onalespib-treated groups. The ^177^Lu-DOTA-M5A treatment also mediated apoptosis in both spheroid models, being most distinct in the LS174T spheroid model. Notably, it is likely that radiotherapy-induced apoptosis might have been even more apparent at later time points than assessed in the present study (82). Nevertheless, even at 24 h posttreatment, the combination therapies exhibited more pronounced cleaved PARP1 expression compared to the corresponding monotreatments and untreated controls in the LS174T spheroid model, indicating a higher level of apoptosis. This is also in line with previous studies, demonstrating that stimulating the TGF-β pathway and MAPK/ERK pathway leads to apoptosis marker overexpression, including X-linked Inhibitor of Apoptosis Protein (XIAP) and caspases, of which all partially end up to cleaved PARP1 (62). To conclude, the molecular assessment of SNU1544 and LS174T 3D tumor lysates indicated that treatments with ^177^Lu-DOTA-M5A and/or onalespib could possibly restore SMAD3 expression in the TGF-β pathway. Moreover, EGFR and HSP70 alterations confirmed HPS90 inhibition in the onalespib-treated samples, and PARP1 analyses confirmed increased apoptosis, with enhanced effects in some combination treatments.

## Conclusion

The ^177^Lu-DOTA-M5A is a promising novel radioconjugate with potential for RIT of CEA-expressing colorectal cancers. Moreover, the combination with onalespib further potentiates the therapeutic effects of ^177^Lu-DOTA-M5A potentially owing to the cooperative effects on the cellular tumor-suppressive pathways RTK and MAPK and increased apoptosis. In the future, combining RIT using ^177^Lu-DOTA-M5A with HSP90 inhibitors may be a feasible therapy approach for metastatic colorectal cancers and for overcoming antitumor treatment resistance, and may have the potential to widen the targetable cancer patient population and increase the remission rates.

## Data Availability Statement

The raw data supporting the conclusions of this article will be made available by the authors without undue reservation.

## Author Contributions

TMS contributed to experimental studies with the focus on stability assays, XTT proliferation assays, binding specificity assays, real-time binding assays, 3D tumor spheroid-related assays, and western blotting, analyzing and interpreting the data and drafting and revising the article. PJ contributed to experimental studies with a focus on optimizing radiolabeling, radiolabeling, analyzing and interpreting the data, and drafting and revising the article. AB contributed to the analysis of real-time binding measurement *via* Ligand Tracer and drafting and revision of the article. FYF contributed to the design of the study and data interpretation and revision of the article. PJY contributed to the experimental studies with the focus on the development of the anti-CEA hT84.66 mAb, DOTA conjugation and purification, and drafting and revision of the article. MN initiated and designed the study, contributed to data analysis and interpretation, and drafted and revised the article. All authors have read and approved the final article.

## Funding

The study was supported by Swedish Cancer Society (CAN 21/1534, CAN 20 0191), the Swedish Research Council (2020-01377), the Swedish Childhood Cancer Fund (PR2020-0023, TJ2021-0072), and Stiftelsen Ulf Lundahls minnesfond.

## Conflict of Interest

Author AB is employed by Ridgeview Instruments AB.

The remaining authors declare that the research was conducted in the absence of any commercial or financial relationships that could be construed as a potential conflict of interest.

## Publisher’s Note

All claims expressed in this article are solely those of the authors and do not necessarily represent those of their affiliated organizations, or those of the publisher, the editors and the reviewers. Any product that may be evaluated in this article, or claim that may be made by its manufacturer, is not guaranteed or endorsed by the publisher.
