# Supplementary figures and images for: In Vitro Characterization of 177Lu-DOTA-M5A Anti-Carcinoembryonic Antigen Humanized Antibody and HSP90 Inhibition for Potentiated Radioimmunotherapy of Colorectal Cancer
Source: Front Oncol. 2022 Mar 31;12:849338. doi: 10.3389/fonc.2022.849338 (PMC9010075; doi:10.3389/fonc.2022.849338)

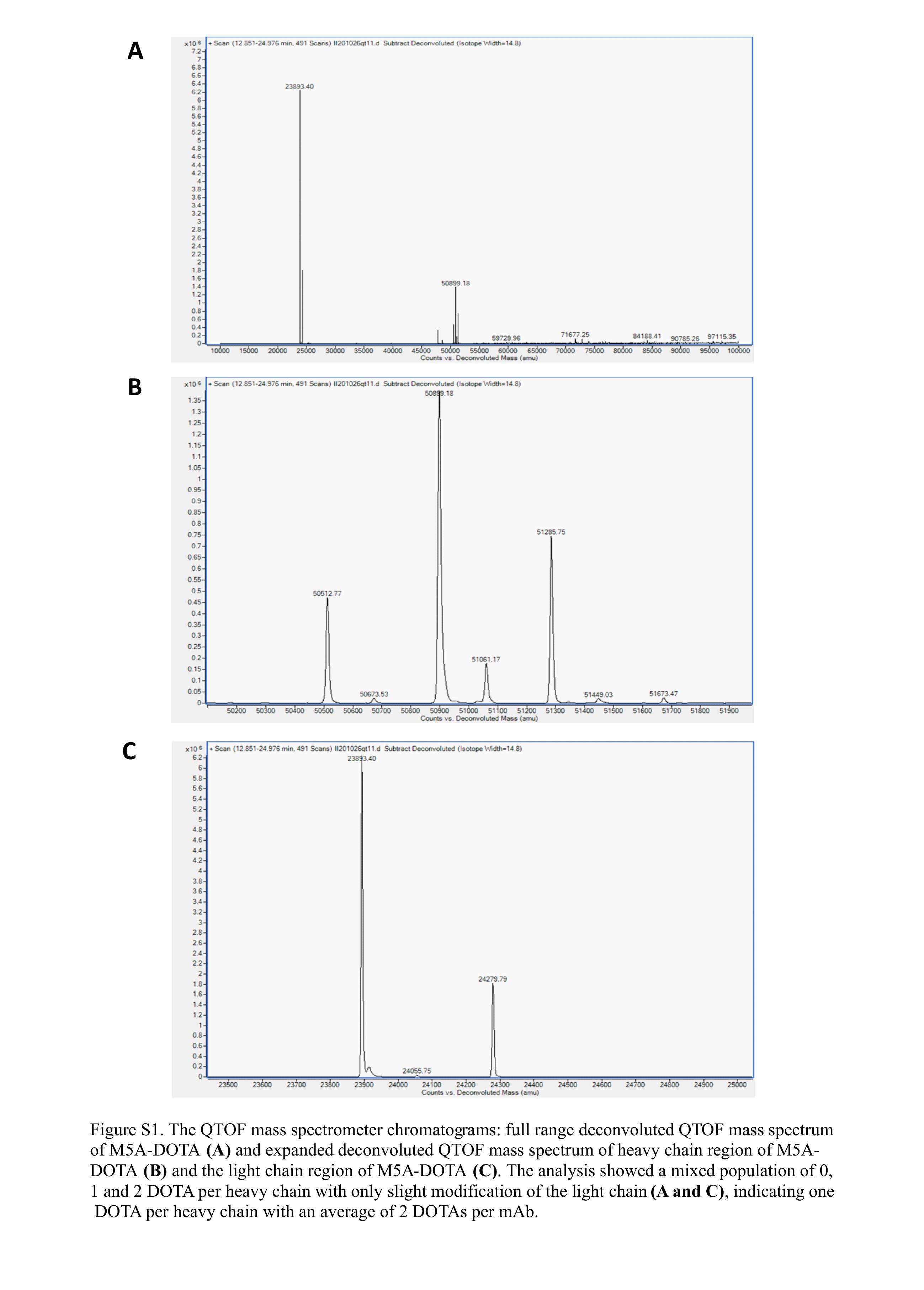

Supplement: Supplementary file 1 [file Image_1.tiff]

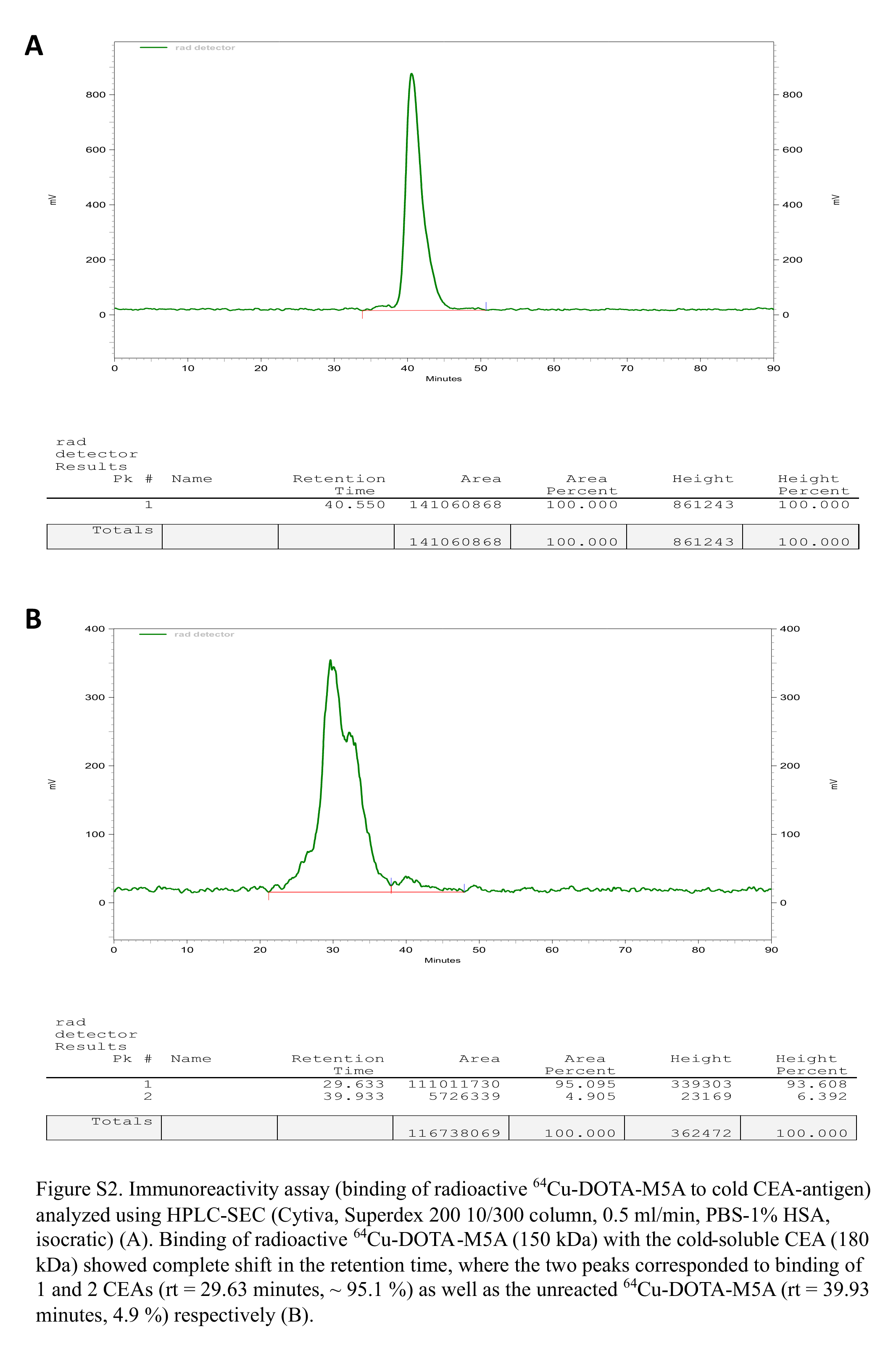

Supplement: Supplementary file 2 [file Image_2.tiff]

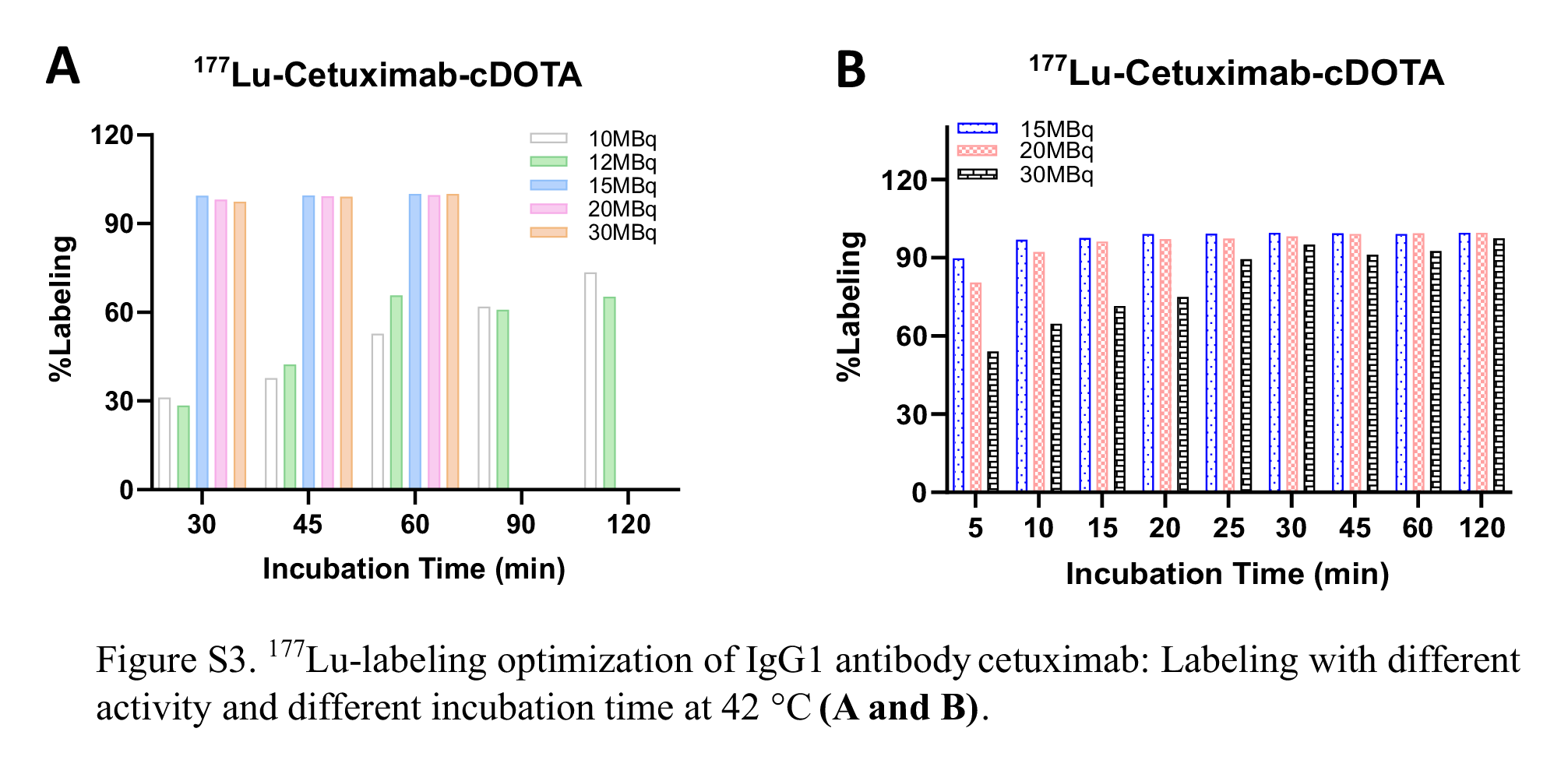

Supplement: Supplementary file 3 [file Image_3.tiff]

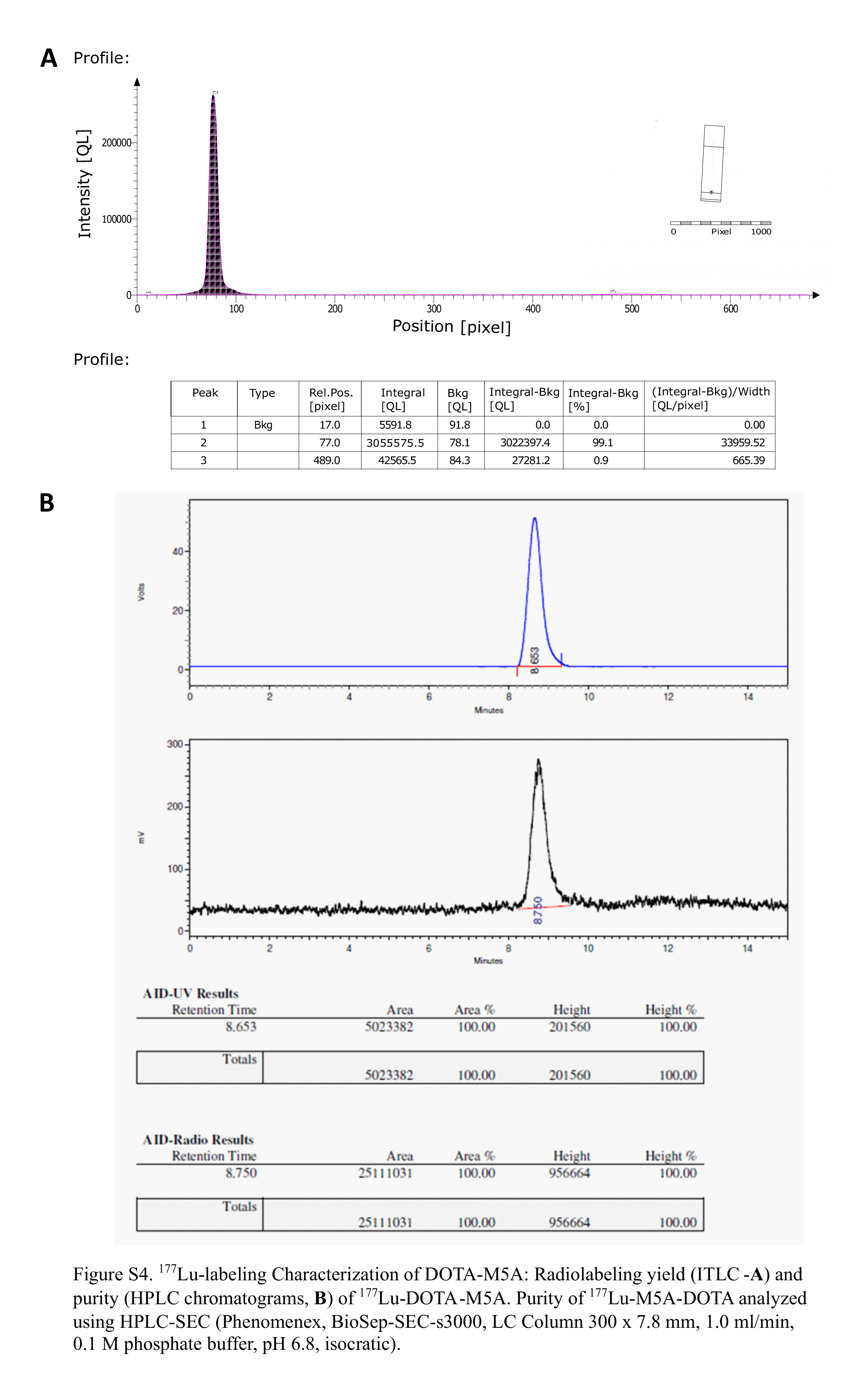

Supplement: Supplementary file 4 [file Image_4.tiff]

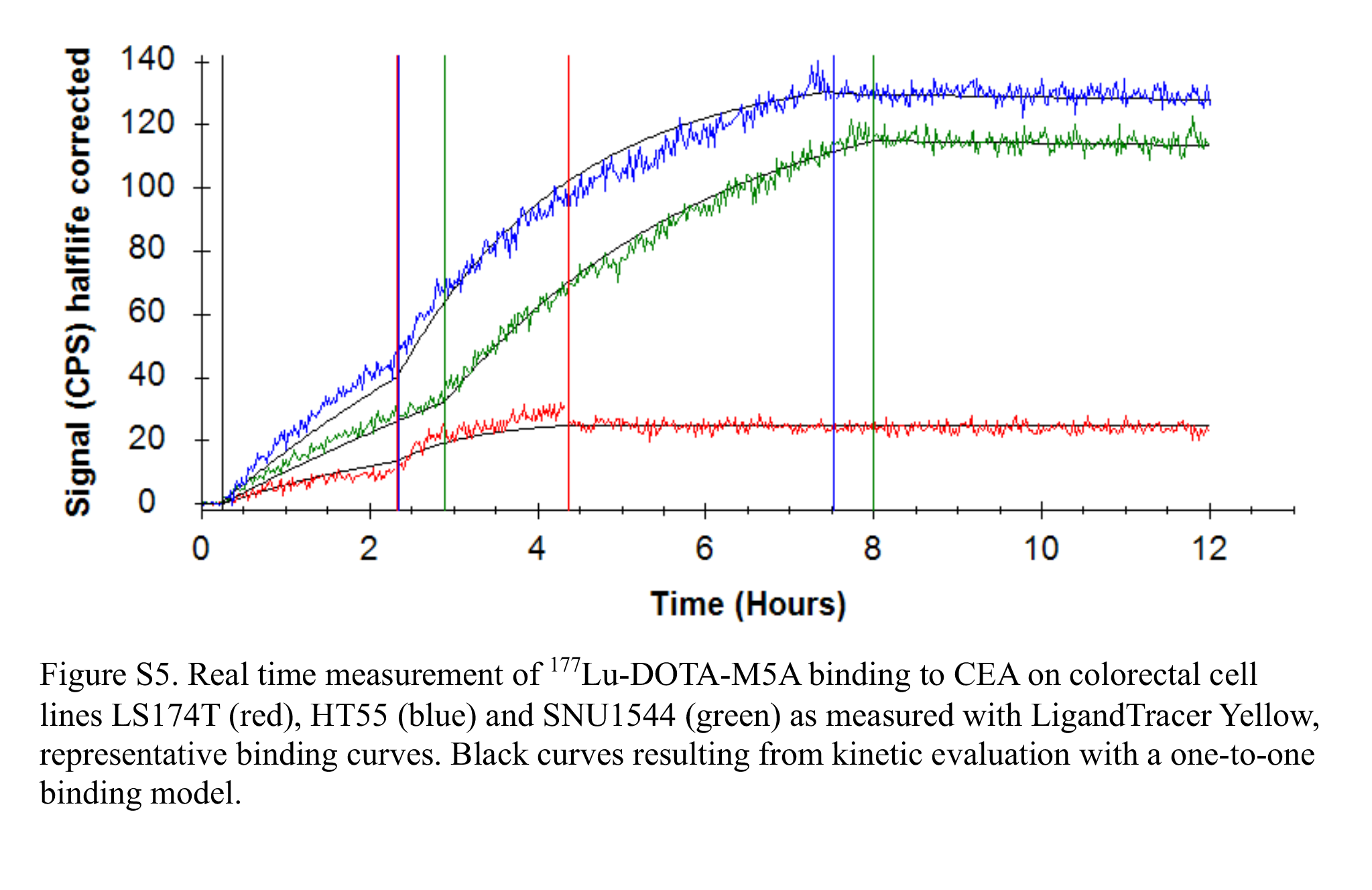

Supplement: Supplementary file 5 [file Image_5.tiff]

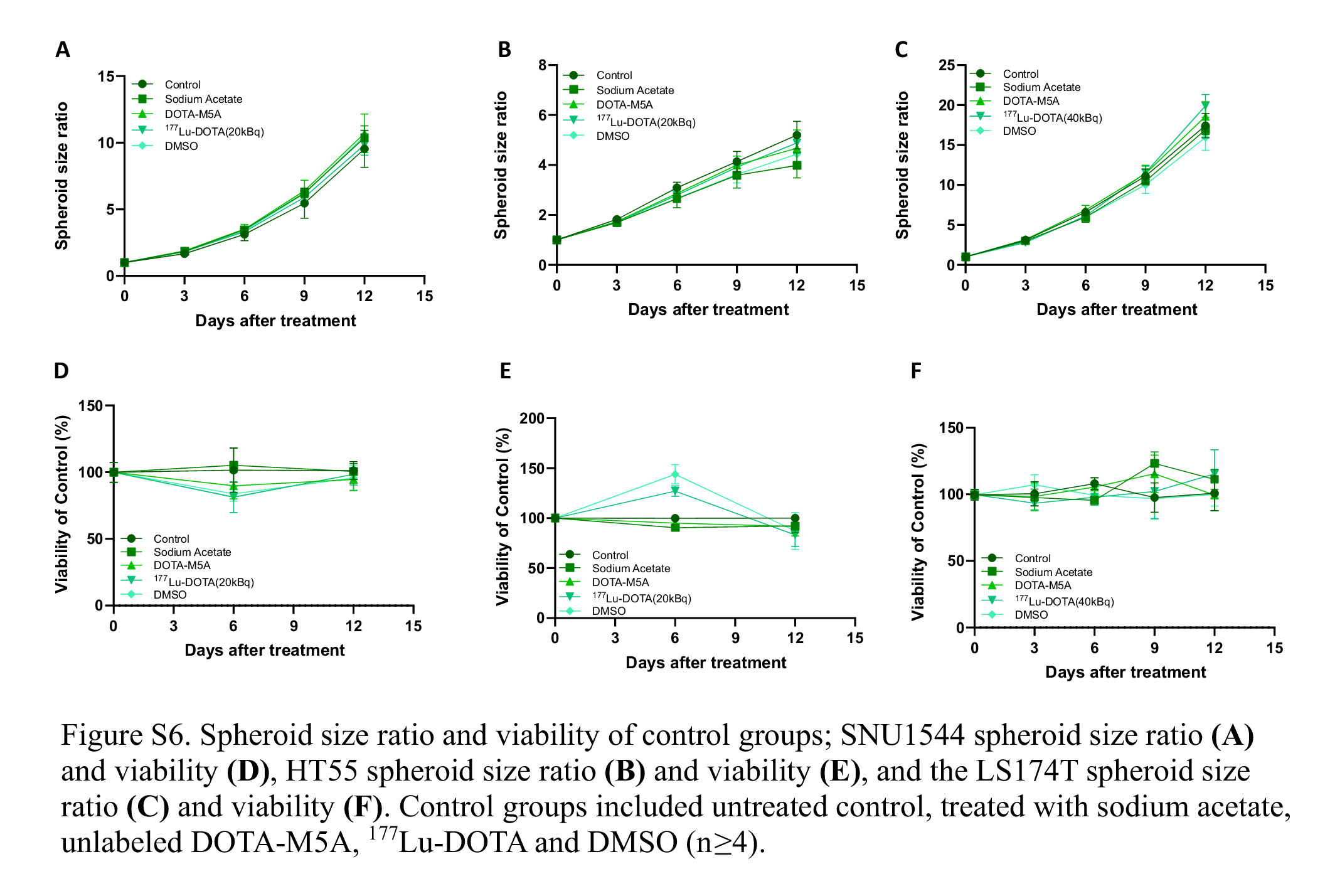

Supplement: Supplementary file 6 [file Image_6.tiff]

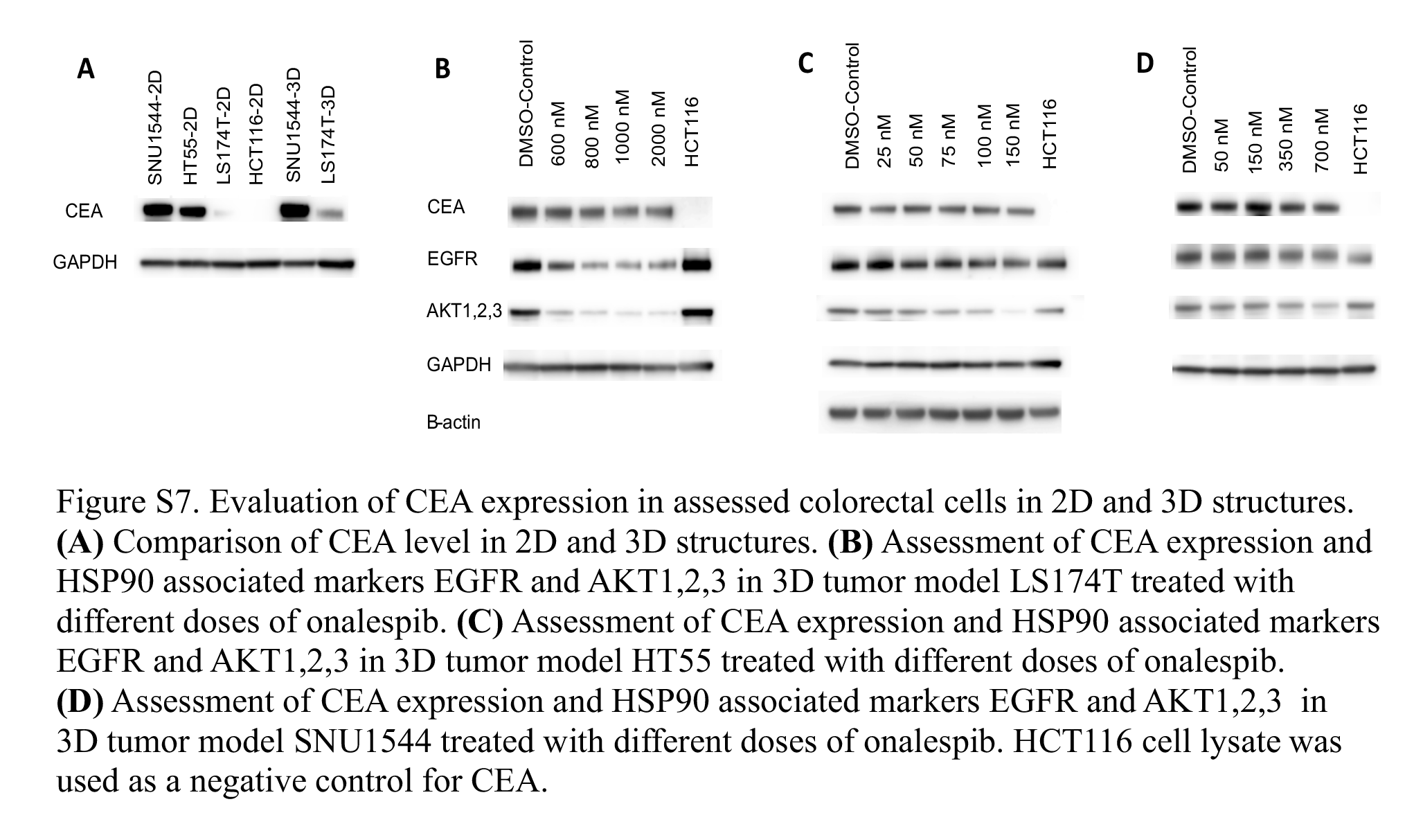

Supplement: Supplementary file 7 [file Image_7.tiff]

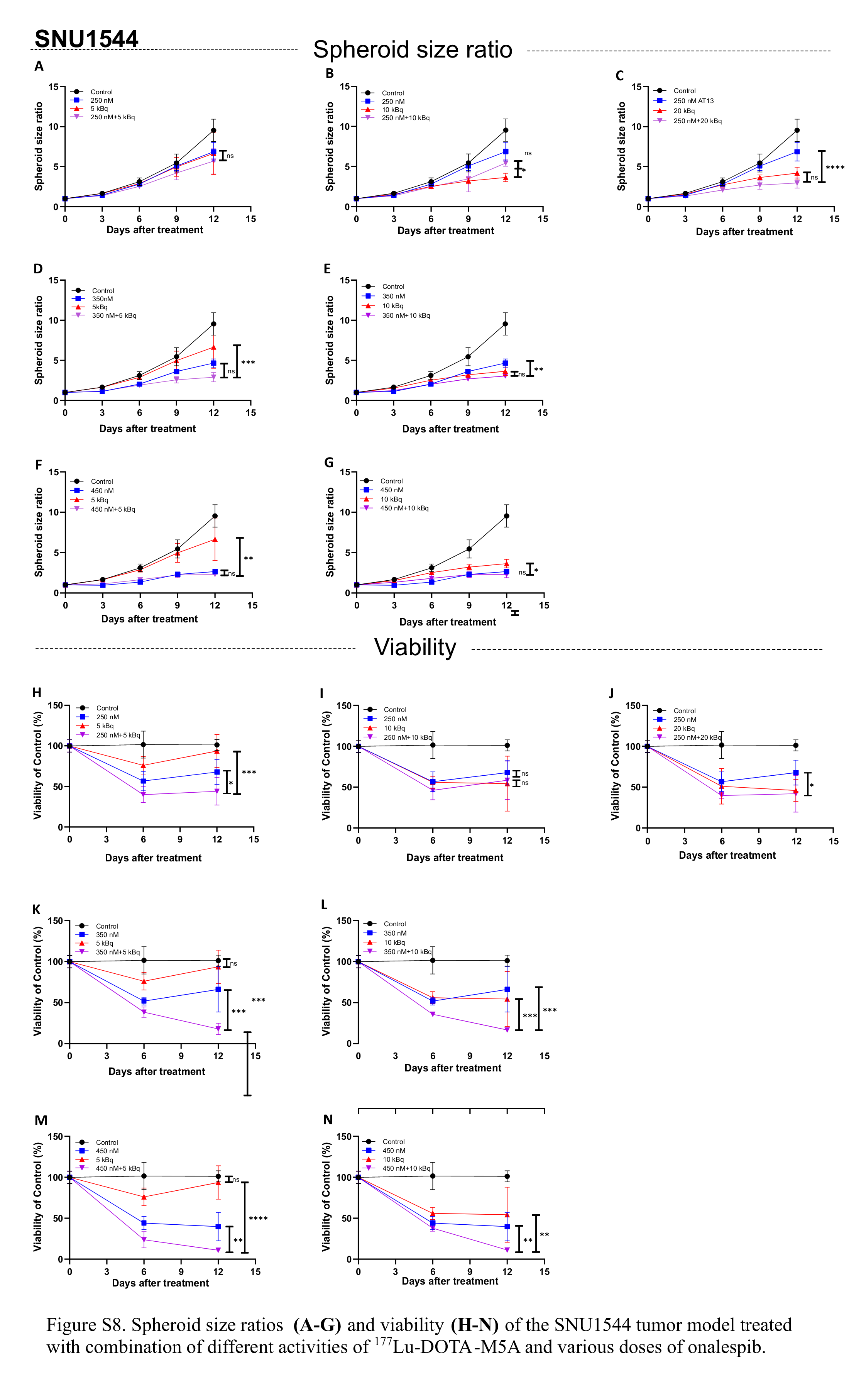

Supplement: Supplementary file 8 [file Image_8.tiff]

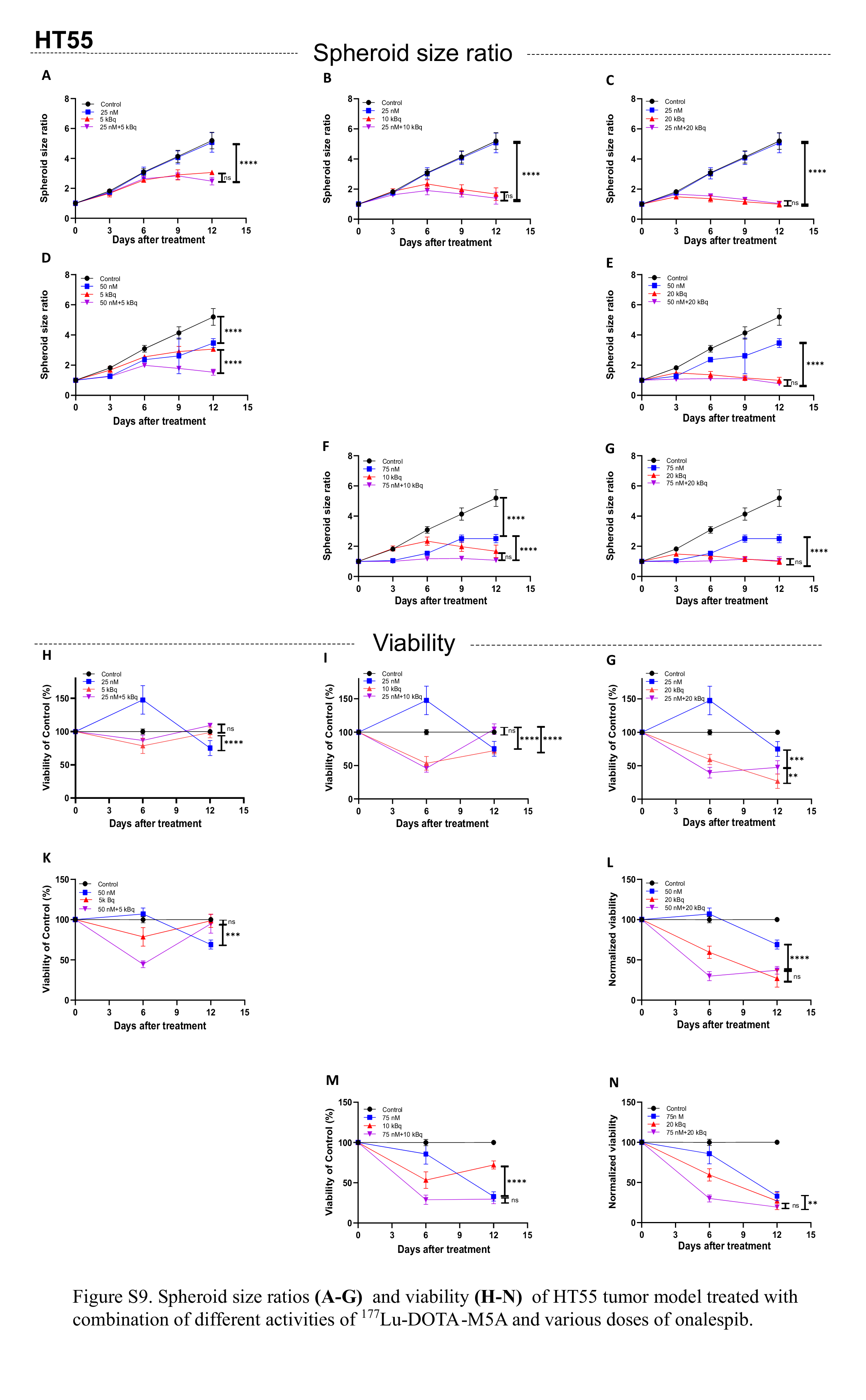

Supplement: Supplementary file 9 [file Image_9.tiff]

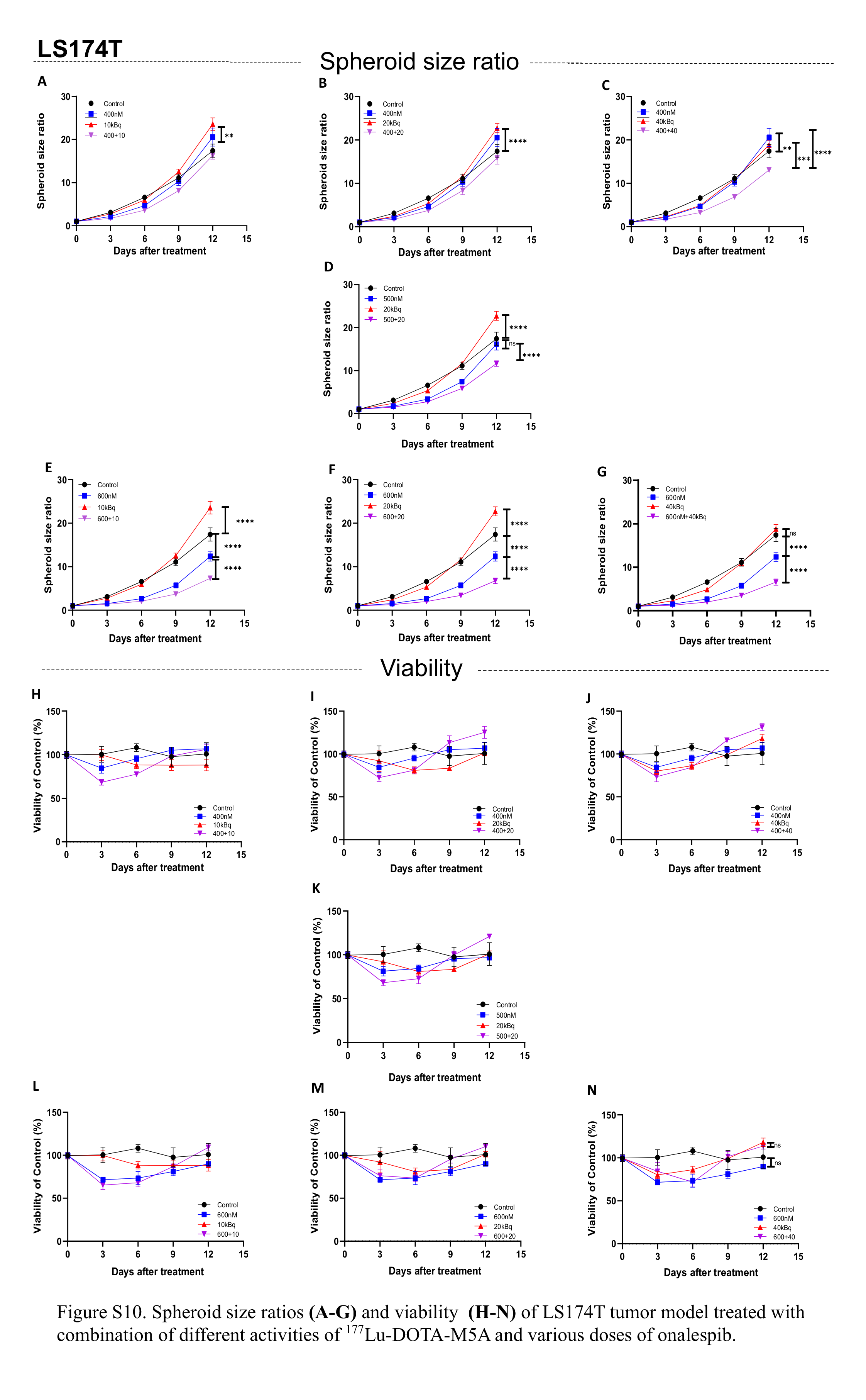

Supplement: Supplementary file 10 [file Image_10.tiff]
